# Supplementary material for: A Phase II trial of Higher RadiOtherapy Dose In The Eradication of early rectal cancer (APHRODITE): protocol for a multicentre, open-label randomised controlled trial
Source: BMJ Open. 2022 Apr 27;12(4):e049119. doi: 10.1136/bmjopen-2021-049119 (PMC9052059; doi:10.1136/bmjopen-2021-049119)
Supplement: Supplementary data [file bmjopen-2021-049119supp001.pdf]

Delete this line, then print on Trust/Hospital headed paper

Participant ID:	Initials:
Date of Birth:	NHS/Hospital Number:
ISRCTN: 161585514	Principal Investigator:

APHRODITE

SAMPLE

PARTICIPANT CONSENT FORM

Please initial each box

1. I confirm that I have read and understand the information sheet for the above study and have had the opportunity to ask questions.
2. I understand that my participation in this study is voluntary and that I am free to withdraw at any time without my medical care or legal rights being affected. I understand that even if I withdraw from the above study, the data and samples collected from me will be used in analysing the results of the study and in some cases further information about any unwanted effects of my treatment may need to be collected by the study team.
3. I understand that my healthcare records may be looked at by authorised individuals from the study team, regulatory bodies or Sponsor in order to check that the study is being carried out correctly.
4. I agree to a copy of this Consent Form being sent to the Clinical Trials Research Unit (CTRU).
5. I will allow any information or results arising from this study to be used for healthcare and/or further medical research upon the understanding that my identity will remain anonymous wherever possible.
6. I will allow any information or results arising from this study to be shared anonymously with researchers at the University of Leeds conducting the study “How do patients make decisions about rectal cancer treatment?” I understand that my identity will remain anonymous.

7. I understand that if during this study my clinical care team determine that I have lost my ability to make my own decisions, no further study intervention will be given. I agree that the information collected up until this point will remain on file and will be included in the analysis. ☐
8. I agree that my GP, or any other doctor treating me, will be notified of my participation in this study. ☐
9. I agree to take part in the study. ☐

The following points are OPTIONAL.

	Yes	No
	Please initial each box	
I give permission for surplus samples from my cancer biopsy that have been stored in the hospital pathology laboratory to be retrieved and used in the future for colorectal cancer research, including genetic research.	☐	☐
I understand that my tissue sample is a 'gift' that may be used in future research that receives ethical approval. I understand that my sample and data collected from it may be shared on a collaborative basis with researchers in the UK and potentially, centres abroad, including outside the European Economic Area.	☐	☐
I give permission that my endoscopy photos, radiotherapy data, CT and MRI scans may be used in future research. I understand that my radiotherapy data scans may be shared on a collaborative basis with researchers in the UK and potentially, centres, abroad, including outside the European Economic Area.	☐	☐

SAMPLE

Patient:

Signature…………………………………………………………………………………

Name(block capitals)……………………………………………………………………………

Date………………………………………………………………………………………

Investigator:

I have explained the study to the above named patient and he/she has indicated his/her willingness to participate.

Signature………………………………………………………………………………

Name(block capitals)……………………………………………………………………………

Date………………………………………………………………………………………

(If used) Witness:

Signature………………………………………………………………………………

Name(block capitals)……………………………………………………………………………

Date………………………………………………………………………………………

(1 copy for patient; 1 for the CTRU; 1 held in patient notes, original stored in Investigator Site File)

SAMPLE
