# Supplementary material for: A Phase II trial of Higher RadiOtherapy Dose In The Eradication of early rectal cancer (APHRODITE): protocol for a multicentre, open-label randomised controlled trial
Source: BMJ Open. 2022 Apr 27;12(4):e049119. doi: 10.1136/bmjopen-2021-049119 (PMC9052059; doi:10.1136/bmjopen-2021-049119)
Supplement: Author's manuscript [file bmjopen-2021-049119.draft_revisions.pdf]

BMJ Open is committed to open peer review. As part of this commitment we make the peer review history of every article we publish publicly available.

When an article is published we post the peer reviewers' comments and the authors' responses online. We also post the versions of the paper that were used during peer review. These are the versions that the peer review comments apply to.

The versions of the paper that follow are the versions that were submitted during the peer review process. They are not the versions of record or the final published versions. They should not be cited or distributed as the published version of this manuscript.

BMJ Open is an open access journal and the full, final, typeset and author-corrected version of record of the manuscript is available on our site with no access controls, subscription charges or pay-per-view fees (http://bmjopen.bmj.com).

If you have any questions on BMJ Open's open peer review process please email info.bmjopen@bmj.com

BMJ Open

A Phase II trial of Higher RadiOtherapy Dose In The Eradication of early rectal cancer (APHRODITE): Protocol for a multicentre open label randomised controlled trial

Journal:	*BMJ Open*
Manuscript ID	bmjopen-2021-049119
Article Type:	Protocol
Date Submitted by the Author:	15-Jan-2021
Complete List of Authors:	Hudson, Eleanor; University of Leeds, Leeds Institute of Clinical Trials Research<br>Noutch, Samantha; University of Leeds, Leeds Institute of Clinical Trials Research<br>Brown, Sarah; University of Leeds, Leeds Institute of Clinical Trials Research<br>Adapala, Ravi; Wrexham Maelor Hospital, Department of Radiology<br>Bach, Simon; Queen Elizabeth Hospital Birmingham, Academic Department of Surgery<br>Burnett, Carole ; St James's University Hospital, Leeds Cancer Centre<br>Burrage, Alwyn; Patient advocate<br>Gilbert, Alexandra; University of Leeds, Leeds Institute of Medical Research at St James's<br>Hawkins, Maria; University College London, Medical Physics and Biomedical Engineering<br>Howard, Debra; Mount Vernon Cancer Centre, National Radiotherapy Trials QA (RTTQA) Group<br>Jefford, Monia; Patient advocate<br>Kochhar, Rohit; The Christie NHS Foundation Trust<br>Saunders, Mark ; The Christie NHS Foundation Trust<br>Seligmann, Jenny; University of Leeds, Leeds Institute of Medical Research at St James's<br>Smith, Alexandra; University of Leeds, Leeds Institute of Clinical Trials Research<br>Teo, Mark; St James's University Hospital, Leeds Cancer Centre<br>Webb, Edward; University of Leeds, Leeds Institute of Health Sciences<br>Webster, Amanda; Mount Vernon Hospital, National Radiotherapy Trials QA (RTTQA) Group<br>West, Nick; University of Leeds, Leeds Institute of Medical Research at St James's<br>Sebag-Montefiore, David ; University of Leeds, Leeds Institute of Medical Research at St James's<br>Gollins, Simon; Glan Clwyd Hospital, North Wales Cancer Treatment Centre<br>Appelt, Ane; University of Leeds, Leeds Institute of Medical Research at St James's
Keywords:	Clinical trials < THERAPEUTICS, Gastrointestinal tumours < ONCOLOGY, RADIOTHERAPY

A Phase II trial of Higher RadiOtherapy Dose In The Eradication of early rectal cancer (APHRODITE): Protocol for a multicentre open label randomised controlled trial

Authors:

Eleanor M Hudson[1], Samantha Noutch[1], Sarah Brown[1], Ravi Adapala[2], Simon P Bach[3], Carole Burnett[4], Alwyn Burrage[5], Alexandra Gilbert[6], Maria Hawkins[7], Debra Howard[8], Monica Jefford[5], Rohit Kochhar[9], Mark Saunders[9], Jenny Seligmann[6], Alexandra Smith[1], Mark Teo[4], Edward JD Webb[10], Amanda Webster[8], Nicholas West[6], David Sebag-Montefiore[6], Simon Gollins[11]*, Ane Appelt[6]*

Shared last authorship

Author affiliation:

[1]Clinical Trials Research Unit, Leeds Institute of Clinical Trials Research, University of Leeds, Leeds, United Kingdom

[2]Department of Radiology, Wrexham Maelor Hospital, Wrexham, United Kingdom

[3]Academic Department of Surgery, Queen Elizabeth Hospital Birmingham, United Kingdom

[4]Leeds Cancer Centre, St James's University Hospital, Leeds, United Kingdom

[5]Patient Advocate, United Kingdom

[6]Leeds Institute of Medical Research at St James's, University of Leeds, Leeds, United Kingdom

[7]Medical Physics and Biomedical Engineering, University College London, London, United Kingdom

[8]National Radiotherapy Trials QA (RTTQA) Group, Mount Vernon Cancer Centre, Middlesex, United Kingdom

[9]The Christie NHS Foundation Trust, Manchester, United Kingdom

[10]Academic Unit of Health Economics, Leeds Institute of Health Sciences, University of Leeds, Leeds, United Kingdom

[11]North Wales Cancer Treatment Centre, Glan Clwyd Hospital, Bodelwyddan, United Kingdom

Corresponding author: Dr. Ane Appelt

A.L.Appelt@leeds.ac.uk

Leeds Institute of Medical Research at St James's,

University of Leeds,

St James's University Hospital,

Harehills

Leeds,

LS9 7LP

+44 113 206 7685 (office)

Word count (excluding title page, abstract, references, figures and tables): 3882

For peer review only

ABSTRACT

Introduction

The standard of care for patients with localised rectal cancer is radical surgery; often combined with pre-operative neoadjuvant (chemo-)radiotherapy. While oncologically effective, this treatment strategy is associated with operative mortality risks, significant morbidity, and stoma formation. An alternative approach is chemoradiotherapy to try to achieve a sustained clinical complete response (cCR). This non-surgical management can be attractive, particularly for patients at high risk of surgical complications. Modern radiotherapy techniques allow increased treatment conformality, enabling increased radiation dose to the tumour while reducing dose to normal tissue. The objective of this trial is to assess if radiotherapy dose escalation increases the cCR rate, with acceptable toxicity, for treatment of early rectal cancer patients unsuitable for radical surgery.

Methods and analysis

APHRODITE is a multicentre open label, randomised controlled phase II trial aiming to recruit 104 participants from 10-12 UK sites. Participants will be allocated with a 2:1 ratio of intervention:control. The intervention is escalated dose radiotherapy (62 Gy to primary tumour, 50.4 Gy to surrounding mesorectum in 28 fractions) using synchronous integrated boost (SIB). The control arm will receive 50.4 Gy to the primary tumour and surrounding mesorectum. Both arms will utilise intensity modulated radiotherapy (IMRT) and daily image guidance, combined with concurrent chemotherapy (Capecitabine, 5-Fluorouracil / Leucovorin or omitted). The primary endpoint is the proportion of participants with cCR at 6-months post start of treatment. Secondary outcomes include early and late toxicities, time to stoma formation, overall survival, and patient reported outcomes (QLQ-C30, QLQ-CR29, LARS questionnaire).

For peer review only

Conclusion

The APHRODITE study will investigate the efficacy and safety of escalated dose radiotherapy for treatment of early stage rectal cancer patients unsuitable for surgery. The results will inform future trials and clinical care.

Trial registration

ISRCTN: 16158514. Date of registry 17th October 2019.

Funding

Yorkshire Cancer Research (YCR).

For peer review only

STRENGTHS AND WEAKNESSES

- APHRODITE is a prospective, multi-centre randomised controlled trial, in contrast to previous studies of organ preservation that have primarily been retrospective, single centre or single arm.
- The trial offers treatment for patients who are not good candidates for (standard) surgical management, thus serving a group of patients often excluded from clinical trials.
- Long course radiotherapy may be intolerable for some very frail patients, for whom short course treatment may be preferential; this study will not assess short course radiotherapy.
- The phase II design is focused on generating preliminary evidence of efficacy; a subsequent phase III trial will be required to evaluate longer-term clinical impact.
- The study has embedded a strong focus on patients and their assessment of outcomes, including patient reported outcome measures (PROMs) and a translational sub-study of patient treatment preferences.

For peer review only

INTRODUCTION

Colorectal cancer is the third most common cancer in the United Kingdom (UK). Each year around 42,000 new cases are diagnosed, with over one quarter within the rectum [1]. The standard of care for patients with localised rectal cancer is radical surgery combined with a selective use of pre-operative radiotherapy and adjuvant chemotherapy. Radical surgery consists of total mesorectal excision (TME) which is an oncologically effective treatment for early stage rectal cancer; only 2% and 12% of patients will experience local or distant failure respectively [2-5]. However, radical surgery is associated with risks of operative mortality, significant morbidity, and the frequent need for stoma.

Mortality following elective major colorectal cancer surgery has fallen significantly in recent years and the 2020 UK National Bowel Cancer Audit Annual Report, relating to 2018-2019, shows overall 90 day mortality of 3% [6]. However, post-operative mortality for rectal cancer increases with age as demonstrated in the Dutch TME trial [7, 8] and is highly dependent on patients' general physical fitness, with significantly increased 30-day mortality in patients with American Society of Anaesthesiologists (ASA) grade III and above physical status [9].

In the 2020 UK National Bowel Cancer Audit [6], 86% of rectal cancer patients had a stoma following major resection, including 78% of patients undergoing anterior resection. Eighteen months later 29% of these patients still had a stoma. Stoma-related morbidity affects at least 50% of patients; with the most common problems including high-output stomas, stoma prolapses, small bowel obstruction and wound infections [10]. Amongst the patients not undergoing radical resection, many are older or frailer, with comorbidities, or those that may struggle to manage a stoma. Surgery is often not a good option for these patients yet there is no standard alternative treatment.

For peer review only

An alternative approach to surgery is the use of radiotherapy with or without chemotherapy (CRT) to try to achieve a sustained clinical complete response (cCR) and potential long-term tumour control. Registration studies of patients with (primarily) locally advanced cancer who have achieved a cCR after neoadjuvant treatment, and opted for non-surgical management, have reported on the oncological safety of this approach [11-13]. A systematic review of 23 cohort studies of patients managed non-surgically following cCR suggested a pooled 2-year local regrowth rate of 21.3% in 575 cCR patients. The vast majority (93.2%) of patients with local regrowth were subsequently managed with salvage surgery [14]. Consequently, a policy of ‘active surveillance’ is increasingly being adopted, for patients in whom post-CRT endoscopy and magnetic resonance imaging (MRI) suggest a cCR.

Most published studies have focused on an ‘opportunistic’ approach in patients achieving a cCR following CRT as part of standard clinical management rather than exploring dedicated organ preservation strategies (planned intent). Only a small minority of locally advanced rectal cancers achieve a cCR with standard CRT, however, changing our focus to earlier cancer, which are more likely to respond to chemoradiotherapy, there is the potential for considerably improving response rates. This opens for studies of dedicated and upfront non-surgical management strategies, using chemoradiotherapy as a definitive treatment.

There is moderate evidence of a radiation dose-response relationship for pathological tumour regression after preoperative (chemo-)radiotherapy [15, 16]. Currently, there is no high-level evidence as to whether dose escalation increases the cCR rate, although selected publications have reported high cCR and organ preservation rates with high-dose chemoradiotherapy for early cancers [17]. The non-randomised nature of these studies is a major limitation, particularly with the inclusion of small and early cancers which are more likely to respond [18]. Additionally, there is limited evidence of the optimum dose level for dose escalation, though experience from anal cancer and preliminary data from two ongoing Danish studies [NCT02438839, NCT04095299] indicate that 62 Gy can be safely delivered to a confined target volume in 2.2Gy per fraction.

For peer review only

Modern radiotherapy techniques, including Intensity Modulated Radiotherapy (IMRT) and volumetric arc therapy (VMAT) with image guidance (IGRT), improve conformality of treated volumes. Theoretically this will reduce early and late toxicity when compared to conventional 3D conformal planning [19]. These advances offer the possibility of increasing the dose to the tumour without excessive morbidity of surrounding normal tissues. Dose escalation with simultaneous integrated boost (SIB) can thus be achieved without significantly altering the dose delivered to the organs at risk, and without extending the overall treatment time.

To further minimise radiation-induced side effects, a risk-adapted target volume can be used. The risk of pelvic lymph node involvement or distal mesorectal nodal involvement is very low for patients with early rectal cancer [20, 21]. Additionally, local recurrences are unlikely to be found above the level of the S2/3 interspace [22]. In early rectal cancer, it is thus doubtful that traditional, large elective treatment volumes are indicated. Therefore, it is reasonable to reduce the target volume to the peritumoral region of the primary tumour and the mesorectum [23, 24].

The APHRODITE phase II study will utilise modern radiotherapy techniques to investigate the efficacy and safety of escalated dose radiotherapy for non-surgical treatment of early stage rectal cancer in patients not suitable for standard surgical management.

METHODS AND ANALYSIS

Design and aim

APHRODITE is a phase II, multi-centre, open-label, randomised-controlled trial of IMRT, with select use of concomitant chemotherapy, in patients with early stage rectal cancer (T1-3bN0, maximum diameter ≤ 4 cm) who are deemed not suitable for radical TME surgery by their multidisciplinary team (MDT). The primary aim is to assess whether radiotherapy dose escalation

For peer review only

increases the cCR rate at 6 months from the start of CRT, compared with standard radiotherapy dose CRT, with acceptable toxicity. Patients may receive full dose, reduced dose or no concominant chemotherapy. A total of 104 eligible patients will be recruited from 10-12 UK radiotherapy sites. Participants will be randomised on a 2:1 basis to receive escalated dose or standard dose chemoradiation. The participant pathway can be seen schematically in Figure 1. The study protocol and this manuscript have been written in accordance with Standard Protocol Items: Recommendations for Interventional Trials (SPIRIT) guidelines.

Trial objectives

The primary objective of APHRODITE is to compare the proportion of participants with a cCR at 6-months from the start of CRT. A composite definition for cCR is used, including digital rectal examination (DRE), rectal endoscopy and pelvic MRI, defined by:

1. No evidence of either mucosal tumour or submucosal swelling on white light endoscopy. A flat white scar remains, with or without telangiectasia.
2. No palpable tumour upon DRE.
3. High resolution pelvic MRI scanning shows either a linear scar only, or dense fibrosis with no obvious tumour signal (mrTRG 1 or 2) [25, 26].

Every effort will be made to ensure trimodality assessment (endoscopy, DRE and MRI). If such is not possible, confirmation should always include a rectal endoscopy and one other (DRE, MRI). The protocol does not require use of biopsy for response evaluation.

Currently research is unclear whether high dose radiotherapy increases the rate of superficial mucosal ulcerations in the presence of a complete primary tumour response. Therefore, an alternative definition of the primary endpoint, with the inclusion of superficial mucosal ulceration classified as cCR, will be explored in a sensitivity analysis.

The secondary objectives of APHRODITE evaluate the safety and tolerability of dose escalation based on:

For peer review only

- Acute toxicities as per Common Terminology Criteria for Adverse Events (CTCAE V.5.0.) (measured weekly during and up to two weeks post end of treatment).
- Treatment compliance (both radiotherapy and, if received, concomitant chemotherapy).
- Toxicities during follow-up as per CTCAE V5.0 (measured up to the 24-month follow-up point).

The longer-term activity of dose escalation will be evaluated based on:

- Time to stoma formation (time from randomisation to stoma formation – colostomy or ileostomy).
- Overall survival (time from randomisation to death from any cause).
- Patient reported outcome measures (PROMs), assessed by the European Organisation for Research and Treatment of Cancer (EORTC) QLQ-C30 [27], EORTC QLQ-CR29 [28], Low Anterior Resection Syndrome (LARS) questionnaire [29] and additional EORTC-QLQ items from the Item Library relevant for organ preservation.

Study population

Eligible patients are those with early stage rectal cancer who are deemed by their MDT not suitable for radical TME surgery because either:

i. The patient is thought to be at increased surgical risk due to specific medical co-morbidity or general frailty.
ii. The patient has marked anxiety at the prospect of having a stoma.
iii. It is anticipated that the patient would have difficulty managing a stoma post-operatively e.g. due to physical problems.

See Table 1 for a full list of inclusion and exclusion criteria.

For peer review only

Table 1– Inclusion and exclusion criteria

**Inclusion criteria**	**Exclusion criteria**
1. Biopsy confirmed adenocarcinoma of the rectum	1. Nodal involvement on MRI (i.e. N1- N2) * or discontinuous tumour deposits (N1c)
2. Age 18 years or over	2. The presence of EMVI discontinuous with the primary tumour on MRI
3. Able to provide written informed consent	3. Involvement of anal intersphincteric plane or external anal sphincter or adjacent organs; or tumour involves/breaches levator ani
4. Patient deemed unsuitable for TME surgical resection, see main text for details	4. Tumour grown through and breached mesorectal fascia
5. Patient suitable for pelvic radiotherapy or chemoradiation: ECOG PS 0-2	5. Signet ring carcinoma or tumours histopathologically containing a neuroendocrine component; or dominant mucinous tumour on MRI
6. Primary tumour staged T1-T3b *, and maximum tumour diameter ≤ 4 cm	6. Undergone an attempt at complete local resection of their cancer
7. No unequivocally involved lymph nodes, NX and N0 eligible *	7. Definite distant metastases (equivocal distant metastases are permitted)
8. Tumour visible on MRI	8. Defunctioning colostomy/ileostomy fashioned
9. Superior aspect of tumour is at or below a horizontal line drawn from the anterior aspect of the S2/3 junction on pre-treatment MRI	9. Previous pelvic radiotherapy; or prior systemic chemotherapy for colorectal cancer
10. For low rectal tumours, superior to the puborectalis sling, the mesorectal fascia or levator is:<br>○ Clear (>1mm from disease to levator ani or mesorectal fascia)<br>○ Or threatened (≤1mm from disease to levator ani or mesorectal fascia)<br>○ Or mesorectal fascia is involved but not breached	10. Prior invasive malignancy unless disease free for a minimum of 3 years (excluding basal cell carcinoma of the skin or other in situ carcinomas)
11. Blood counts fulfilling:<br>○ Estimated creatinine clearance ≥ 50 ml/min<br>○ Absolute neutrophil count > 1.5 x 109/l; platelets > 100 x 109/l<br>○ Serum transaminase concentration < 3 x Upper Limit Normal (ULN)<br>Bilirubin concentration < 1.5 x ULN	11. Women who are pregnant, breastfeeding or of child bearing potential and unwilling to use contraceptives

** TNM staging as per UICC 8th Edition. EMVI = extramural venous invasion.*

For peer review only

Randomisation and recruitment

A computer-generated minimisation program incorporating a random element will be used to allocate patients on a 2:1 basis to receive escalated dose or standard dose chemoradiation, ensuring arms are well-balanced for the following factors:

- Randomising site
- T-stage (<T3 vs. T3)
- Chemotherapy use (either 100% or 75% dose) vs. no chemotherapy use

Randomisation will be performed centrally via the University of Leeds Clinical Trials Research Unit (CTRU) automated 24-hour randomisation system. The registration and randomisation process will be instigated by on-site research staff; patient consent form must be attained prior to registration.

Recruitment will take place over 2 years, with an estimated monthly recruitment rate of 4-6 patients per month. APHRODITE opened in February 2020, with expected recruitment completion by winter 2022. The study has and will be presented at national and international meetings, increasing clinical awareness. A trial website and dedicated twitter account will be utilised to further increase exposure, highlighting the trial population criteria to treating clinicians and MDT members. Based on preliminary feedback from clinicians and patient representatives in the study design phase, the 2:1 allocation between the intervention and control arms will make recruitment attractive for patients with limited standard treatment options.

Sample size

The target sample size is 104 participants (70 experimental: 34 control), based on a two-group chi-squared test of equal proportions, with continuity correction. Assuming a control arm cCR rate of 35% (Dossa 2017), using a one-sided test at 20% significance, 104 participants (incorporating a 5% loss to follow-up) will provide 80% power to detect an absolute difference of 20% (to 55%)

For peer review only

between arms. Data suggests potential differences as great as 25% (in favour of the experimental arm) (Appelt 2013, Appelt and Jakobsen 2015, Appelt 2015).

An inflated type I error rate of 20% is used as the aim is to show preliminary evidence of activity (Rubinstein 2005, Korn 2012). The direct comparison with the control avoids the risk of incorrect decision making due to uncertain historical control data (Sargent 2009).

Treatment regimen

Intervention arm: the primary tumour will receive 62 Gy in 28 fractions, with 50.4Gy delivered to the surrounding elective mesorectal volume, using a simultaneously integrated boost.

Control arm: the primary tumour and elective mesorectal volume will receive a uniform 50.4Gy in 28 fractions (pre-operation standard dose).

A reduced mesorectum-only elective volume will be used, as appropriate for early stage disease at low risk of nodal failure. All treatment will be planned and delivered using IMRT or VMAT, with extensive radiotherapy guidelines, based on the STAR-TREC trial target definition and treatment planning [23, 24]. Daily image guidance with cone beam CT will be mandatory and promotes precise treatment delivery; essential as the mesorectum and tumour exhibit significant changes in day-to-day position [7, 22]. See Figure 2 for an example treatment plan.

Concurrent chemotherapy will be used during CRT, either as single agent oral capecitabine given twice per day Monday-Friday on the days of radiotherapy throughout the course of radiotherapy, or alternatively as intravenous 5-Fluorouracil/Leucovorin delivered once per day concurrent with fractions 1-5 and 20-25 of radiotherapy (weeks 1 and 5). If the treating clinician decides a patient is not fit to receive the full (100%) dose of chemotherapy, due to co-morbidities, or general frailty, they have the options to treat at 75% dose, or omit chemotherapy (radiotherapy alone). The intended chemotherapy treatment option must be declared for each patient *prior* to randomisation.

For peer review only

Trial assessments and follow-up

All willing patients will be assessed prior to randomisation to confirm full eligibility (Table 1). Recruited participants will be assessed at various timepoints (Table 2).

Table 2 – Trial schedule of assessment

	Early assessments			Treatment		Follow-up assessment (measured post CRT)					
	Eligibility	Baseline	Pre-treat.	On treat.	End of treat.	2w	3m	6 m	9 m	12 m	24 m
Informed consent	X										
Histopathology	X										
Performance status	X		X		X	X	X	X	X	X	X
Bloods	X		X	X	X						
Pregnancy screening	X										
ECG		X									
MRI (pelvis)	X						X	X	Local schedule		
CT (chest, abdo, pelvis)	X						Local standard schedule				
Flexible sigmoidoscopy	X						X	X		X	
Digital rectal examination	X						X	X	X	X	X
Clinical assessment	X			X	X		X	X	X	X	X
Toxicity (CTCAE)		X		X	X	X	X	X	X	X	X
PROMs		X			X		X	X	X	X	X

For further detail of the participation flow see trial schema (Figure 1).

Adverse Reaction (ARs), Serious Adverse Reactions (SARs), Related Unexpected Serious Adverse Events (RUSAEs) and Serious Adverse Events of Interest (SAEoI) will be collected from randomisation until the 24-month follow-up point. SAEoI are events identified by the research team that need additional monitoring; including angina/myocardial infarction, pulmonary embolism, serious skin reactions.

For peer review only

297 Data will be collected from all randomised participants, irrespective of treatment compliance. Where possible, endpoint and PROMs data will be collected from withdrawn participants who do not withdraw consent from further data collection.

Treatment and response assessment quality assurance

The trial will implement robust quality assurance of radiotherapy treatment and the primary endpoint assessments. The radiotherapy quality assurance (QA) programme will be implemented by the National Radiotherapy Trials Quality Assurance Group to ensure treatment is planned and delivered according to the trial protocol. All radiotherapy centres will complete pre-treatment benchmark exercises. Anonymised pre-treatment, 3-month and 6-month endoscopic photographs and MRI scans will be collected for all patients, with the first 2 patients from each site reviewed centrally. Trial Management Group (TMG) specialist members will ensure the inclusion/exclusion criteria have been met (baseline scan) and response has been correctly reported (3 and 6 months), providing feedback where necessary. Further review may be performed in batches.

Statistical analysis

A full statistical analysis plan will be written before any analyses are undertaken. No formal interim analyses are planned within the trial.

The primary endpoint will be assessed on an *Intention to treat (ITT) population*; all randomised participants, grouped by the treatment arm to which they were randomised (regardless of ineligibility, non-compliance or withdrawal).

A multivariable logistic regression model, adjusted for the minimisation factors (excluding site), will be used to compare the proportion of participants achieving a cCR at 6-months. Significance testing will be 1-sided at the 20% level. The treatment effect will be presented as an odds ratio with a two-sided 60% confidence interval (equivalent to a 1-sided 80% lower confidence limit).

For peer review only

Patterns of missing data and imputation methods (including multiple imputation) will be explored, if appropriate. A sensitivity analysis will be performed using the alternative definition of cCR.

All secondary analyses will use a 5% significance level and a two-sided confidence interval, where appropriate. Safety data will be presented on the *safety population*; participants are summarised on the treatment received regardless of randomisation. All other data will be summarised on an ITT population.

Summary statistics will be presented for toxicities on the number and proportion of participants experiencing ARs, SARs, RUSAEs, and SAEoI. Treatment compliance to the allocated radiotherapy (+/- chemotherapy) will be monitored and presented, including summaries for delays, dose modifications and discontinuation. PROMs will be summarised and reported using standard guidelines [29-31]. Mixed effects models will be used to assess differences between treatment arms. Cumulative incidence will be used to compare the time to stoma formation between treatment arms. Overall survival will be compared using the Cox Proportional Hazards model. Kaplan-Meier curves and survival estimates with corresponding confidence intervals will also be presented.

Trial organisation

Trial coordination, data management and statistical analysis will be directed and conducted by the trial specific project team at the University of Leeds CTRU. Trial supervision will be established according to the principles of Good Clinical Practice and in line with the relevant Research Governance Framework within the UK and CTRU standard operating procedures.

Data collection and management

Participating sites will record participant data on trial-specific case report forms and submit them to the CTRU. Participants will complete PROMs on the relevant paper questionnaire forms at the

For peer review only

time of their clinic appointment. All trial data will be entered, validated and stored securely by CTRU. Missing and discrepant data will be flagged and additional data validations raised, as appropriate, from the CTRU data management team. Missing data (except PROMs) will be followed-up until received, or confirmed as not available. Data items regarding consent, patient safety and the primary endpoint will be subject to manual priority checking.

Data will be stored securely at Leeds CTRU. Only CTRU will have access to the data, prior to analysis and release of trial results. Upon completion of the trial, data will be stored in the sponsor archiving facility for a minimum of 15 years. After the final trial results have been published, interested researchers may contact the TMG and CTRU to request access to relevant data. Any requests will be reviewed by the TMG.

Trial monitoring

The TMG will provide ongoing clinical, practical and statistical advice on trial-related matters. The trial will be overseen by an independent Trial Steering Committee (TSC) and Data Monitoring and Ethics Committee (DMEC). The DMEC comprises two clinical oncologists and one statistician. They will review and monitor accumulating interim safety data and unblinded reports, at least annually. Their role is to protect the safety of the participants and maintain the research integrity of the study, advising the TSC on trial developments, including advice on trial continuation. A trial-specific DMEC charter has been developed in line with DAMOCLES [32, 33]. The sponsor and TSC have ultimate oversight over the conduct and continuation of the trial.

Patient preference Sub-study

Dose escalation is associated with a greater probability of tumour control at the cost of greater risk of toxicity, yet little information exists as to what risks patients are willing to accept for a better chance of tumour control. The APHRODITE patient preference study (Chief Investigator, Dr Edward Webb), to be conducted alongside the main trial, aims to address this knowledge gap.

For peer review only

The patient preference sub-study uses a well-established survey technique called a discrete choice experiment (DCE) [34]. DCEs have a simple response format in which patients make a series of choices between hypothetical treatments characterised by attributes, such as probability of tumour control after two years and the risk of side effects [35]. The attribute levels change in each question, allowing the trade-offs participants make to be analysed [36]. The survey has been developed using qualitative methods in line with good practice recommendations [37]. Analysis of responses will quantify maximum acceptable risk of toxicity for a given probability of tumour control, as well as their preferences for process attributes, such as the number of treatment sessions and after-treatment support. Participants will complete the survey before treatment and six months after treatment. This will allow us to assess whether preferences are systematically influenced by treatment experience.

The sub-study plans to recruit 100-200 participants from sites participating in the APHRODITE trial, including APHRODITE participants, patients who decline APHRODITE participation, in addition to patients who are being managed non-surgically but do not meet the trial inclusion/exclusion criteria. Separate ethical approval has been obtained for the patient preference study from an NHS research ethics committee (reference 19/NE/0249).

Sample collection

Pre-treatment routine diagnostic formalin fixed paraffin embedded tumour biopsy tissue samples will be collected on all patients and sent to the University of Leeds, for storage. Consent will be taken for their use in future translational research. The routine glass Haematoxylin & Eosin stained slides used for diagnosis will be scanned to create a digital pathology resource for QA of the trial and support translational research, including research examining biomarkers of radiosensitivity and radioresistance.

For peer review only

ETHICS AND DISSEMINATION

The trial obtained ethical approval from North West Greater Manchester East research ethic committee (reference no. 19/NW/0565) and is registered in the ISRCTN registry (registration number 16158514). Confirmed by the UK Medicines and Healthcare products Regulation Agency, APHRODITE is not a trial of an investigational medicinal product (non-CTIMP). The trial is currently adhering to protocol version 2.0 (approved 31/03/2020).

The primary endpoint and early toxicity data will be analysed and reported when the last participant reaches 6-months post start of treatment. Further analyses will be conducted when all patients have reached 24-months follow-up. The final trial publication will be written by the TMG, and will adhere to ICMJE guidelines.

DISCUSSION

There is currently a lack of prospective trials of dedicated non-surgical management strategies in rectal cancer. It is therefore unclear whether increased radiotherapy dose may contribute to short-term tumour response, long-term local control and organ preservation. In particular, the lack of randomised data from the early phase setting has made it difficult to elucidate whether the high response rates observed in phase II trials are due to patient selection or treatment. The APHRODITE trial will provide high-level evidence whether dose escalation increases the cCR rate when directly compared to standard dose. Further studies will be required to provide evidence for whether a higher radiotherapy dose can provide improved long-term local control without surgery.

Other ongoing studies are examining dedicated organ preservation strategies for early rectal cancer. The STAR-TReC phase III trial (ISRCTN14240288) randomises patients with early (T1-3bN0) cancer with a preference for organ preservation between short- and long-course standard

For peer review only

(chemo-)radiotherapy, with the aim of non-surgical management or local excision only. Both trials remain complimentary: STAR TREC uses standard radiotherapy in a patient population suitable for surgery, while APHRODITE will assess intensified treatment in an alternative patient group. Three other studies of radiotherapy dose escalation strategies for organ preservation are ongoing: The international OPERA phase III trial (NCT02505750) randomises patients between moderately dose escalated CRT (54Gy) and standard dose CRT (45Gy) followed by Papillon contact X-ray therapy (90Gy in 3 fractions); the single-centre randomised phase II MORPHEUS trial (NCT03051464) randomises patients between moderately dose escalated CRT (54Gy) and CRT (45Gy) followed by brachytherapy (30Gy in 3 fractions); and the Danish multi-centre WW3 phase II trial (NCT04095299) randomises patients between dose-escalated CRT (62Gy) and standard dose CRT (50.4Gy). All these studies require suitability for surgery, and the first two depend on access to specialised treatment equipment. The APHRODITE study thus fills a clear research gap in the current international trial portfolio.

APHRODITE has been developed with patient and public involvement (PPI) representatives from the earliest stages. Multiple aspects of both trial design and delivery have been shaped in collaboration with our PPI representatives. Upon their suggestion, APHRODITE has a patient-facing trial website, and a patient newsletter will be issued at regular intervals. Trial feedback and communication with participants has been highlighted as a key importance.

Studies of non-surgical management and organ preservation for rectal cancer have focused on clinical endpoints, e.g. cCR rate, stoma rate, and overall survival, as well as the clinicians' interpretation of patient outcome. There is a paucity of toxicity data including PROMs following nonsurgical management, particularly in a patient population usually excluded from clinical trials. The APHRODITE study will prospectively measure toxicity and PROMs, providing a valuable and unique dataset.

For peer review only

At present, there is a lack of understanding of how patients weigh and prioritise different potential outcomes, including the balance between efficacy and toxicity, in the setting of non-surgical rectal cancer treatment. The decision regarding which severity of treatment-related toxicities is acceptable for a given organ preservation strategy has previously been evaluated purely by clinicians. Two small studies examined patient preferences for organ-preserving and surgical treatment approaches [38, 39]. However, no studies have focused specifically on patient attitudes to treatment characteristics and outcomes following non-surgical management. The APHRODITE patient preference sub-study will help inform future dose escalation studies and help non-surgical management of rectal cancer to better reflect patient perspectives.

CONCLUSION

The APHRODITE trial will provide valuable, high quality, patient-centred evidence for escalated dose radiotherapy in rectal cancer patients not suitable for radical surgery, addressing an important research gap.

For peer review only

AUTHOR CONTRIBUTIONS

Contributors to the conception and design: AA, DSM, SB, SG.
Development of the protocol, trial documents and patient information sheet: AA, AB, AG, AW, DSM, DH, EH, JS, MJ, NW, RA, RK, SB, SG, SN.
Writing manuscript: EH, AA.
Reviewed manscript: AB, AG, AW, DSM, EW, MH, MT, NW, SB, SPB, SG.
All authors read and approved the final manuscript.

FUNDING, SPONSOR AND COORDINATION

APHRODITE is supported by Yorkshire Cancer Research (award number L411). The study is coordinated by Leeds CTRU and sponsored by University of Leeds (GA18/118305). For enquires trial contact *email: APHRODITE@leeds.ac.uk.* Trial website: https://ctru.leeds.ac.uk/aphrodite/.

Both the sponsor and funder have no contribution to the study design and concept; collection, management, analysis, and interpretation of data; the writing of and decision to submit reports for publication. The sponsor and funder will review all manuscripts prior to publication.

COMPETING INTERESTS

EW reports grants from Yorkshire Cancer Research, during the conduct of the study.
MS reports personal fees from Servier, personal fees from Amgen, personal fees from Marck, outside the submitted work.
NW reports grants from Yorkshire Cancer Research, during the conduct of the study; grants from Cancer Research UK, outside the submitted work.

For peer review only

EXCLUSIVE LICENCE

I, the Submitting Author has the right to grant and does grant on behalf of all authors of the Work (as defined in the below author licence), an exclusive licence and/or a non-exclusive licence for contributions from authors who are: i) UK Crown employees; ii) where BMJ has agreed a CC-BY licence shall apply, and/or iii) in accordance with the terms applicable for US Federal Government officers or employees acting as part of their official duties; on a worldwide, perpetual, irrevocable, royalty-free basis to BMJ Publishing Group Ltd ("BMJ") its licensees and where the relevant Journal is co-owned by BMJ to the co-owners of the Journal, to publish the Work in BMJ Open and any other BMJ products and to exploit all rights, as set out in our licence.

The Submitting Author accepts and understands that any supply made under these terms is made by BMJ to the Submitting Author unless you are acting as an employee on behalf of your employer or a postgraduate student of an affiliated institution which is paying any applicable article publishing charge ("APC") for Open Access articles. Where the Submitting Author wishes to make the Work available on an Open Access basis (and intends to pay the relevant APC), the terms of reuse of such Open Access shall be governed by a Creative Commons licence – details of these licences and which Creative Commons licence will apply to this Work are set out in our licence referred to above.

For peer review only

FIGURE LEGEND: Figure 1 - *Trial Schema*

FIGURE LEGEND: Figure 2 - *Example treatment plans for the control arm (top panels, uniform 50.4 Gy in 28 fractions to the primary tumour and elective mesorectal volume); and intervention arm (lower panels, 62 Gy in 28 fractions to the primary tumour, 50.4Gy to the elective mesorectal volume), for a single patient.*

REFERENCES

1. CRUK. *Bowel cancer statistics*. 2017 [cited 2020; Available from: https://www.cancerresearchuk.org/health-professional/cancer-statistics/incidence.
2. Bentrem, D.J., et al., *T1 adenocarcinoma of the rectum: transanal excision or radical surgery?* Annals of surgery, 2005. **242**(4): p. 472.
3. Endreseth, B.H., et al., *Transanal excision vs. major surgery for T1 rectal cancer.* Diseases of the colon & rectum, 2005. **48**(7): p. 1380-1388.
4. Peeters, K.C., et al., *The TME trial after a median follow-up of 6 years: increased local control but no survival benefit in irradiated patients with resectable rectal carcinoma.* Annals of surgery, 2007. **246**(5): p. 693-701.
5. Sebag-Montefiore, D., et al., *Preoperative radiotherapy versus selective postoperative chemoradiotherapy in patients with rectal cancer (MRC CR07 and NCIC-CTG C016): a multicentre, randomised trial.* The Lancet, 2009. **373**(9666): p. 811-820.
6. *National Bowel Cancer Audit Annual Report 2020*. 2020.
7. Kapiteijn, E., et al., *Preoperative Radiotherapy Combined with Total Mesorectal Excision for Resectable Rectal Cancer.* New England Journal of Medicine, 2001. **345**(9): p. 638-646.
8. Tekkis, P.P., et al., *Operative mortality in colorectal cancer: prospective national study.* Bmj, 2003. **327**(7425): p. 1196-1201.
9. Rutten, H.J., et al., *Controversies of total mesorectal excision for rectal cancer in elderly patients.* The lancet oncology, 2008. **9**(5): p. 494-501.
10. Bakx, R., et al., *Morbidity of temporary loop ileostomies.* Digestive surgery, 2004. **21**(4): p. 277-281.
11. Habr-Gama, A., et al., *Complete clinical response after neoadjuvant chemoradiation therapy for distal rectal cancer: characterization of clinical and endoscopic findings for standardization.* Diseases of the Colon & Rectum, 2010. **53**(12): p. 1692-1698.

For peer review only

12. Van Der Valk, M.J., et al., *Long-term outcomes of clinical complete responders after neoadjuvant treatment for rectal cancer in the International Watch & Wait Database (IWWD): an international multicentre registry study.* The Lancet, 2018. **391**(10139): p. 2537-2545.
13. Renehan, A.G., et al., *Watch-and-wait approach versus surgical resection after chemoradiotherapy for patients with rectal cancer (the OnCoRe project): a propensity-score matched cohort analysis.* The Lancet Oncology, 2016. **17**(2): p. 174-183.
14. Sammour, T., et al., *Nonoperative management or 'watch and wait'for rectal cancer with complete clinical response after neoadjuvant chemoradiotherapy: a critical appraisal.* Annals of surgical oncology, 2017. **24**(7): p. 1904-1915.
15. Appelt, A.L., et al., *Radiation dose-response model for locally advanced rectal cancer after preoperative chemoradiation therapy.* International Journal of Radiation Oncology* Biology* Physics, 2013. **85**(1): p. 74-80.
16. Valentini, V., et al., *The INTERACT Trial: Long-term results of a randomised trial on preoperative capecitabine-based radiochemotherapy intensified by concomitant boost or oxaliplatin, for cT2 (distal)–cT3 rectal cancer.* Radiotherapy and Oncology, 2019. **134**: p. 110-118.
17. Appelt, A.L., et al., *High-dose chemoradiotherapy and watchful waiting for distal rectal cancer: a prospective observational study.* The lancet oncology, 2015. **16**(8): p. 919-927.
18. Maas, M., et al., *Long-term outcome in patients with a pathological complete response after chemoradiation for rectal cancer: a pooled analysis of individual patient data.* The lancet oncology, 2010. **11**(9): p. 835-844.
19. Appelt, A.L. and D. Sebag-Montefiore, *Technological advances in radiotherapy of rectal cancer: opportunities and challenges.* Current opinion in oncology, 2016. **28**(4): p. 353-358.

For peer review only

20. Roels, S., et al., *Definition and delineation of the clinical target volume for rectal cancer.* International Journal of Radiation Oncology* Biology* Physics, 2006. **65**(4): p. 1129-1142.

21. Leibold, T., et al., *Prognostic implications of the distribution of lymph node metastases in rectal cancer after neoadjuvant chemoradiotherapy.* Journal of clinical oncology, 2008. **26**(13): p. 2106-2111.

22. Nijkamp, J., et al., *Three-dimensional analysis of recurrence patterns in rectal cancer: the cranial border in hypofractionated preoperative radiotherapy can be lowered.* International Journal of Radiation Oncology* Biology* Physics, 2011. **80**(1): p. 103-110.

23. Appelt, A.L., et al., *Robust dose planning objectives for mesorectal radiotherapy of early stage rectal cancer–A multicentre dose planning study.* Technical Innovations & Patient Support in Radiation Oncology, 2019. **11**: p. 14-21.

24. Peters, F.P., et al., *Mesorectal radiotherapy for early stage rectal cancer: A novel target volume.* Clinical and Translational Radiation Oncology, 2020. **21**: p. 104-111.

25. Patel, U.B., et al., *Magnetic resonance imaging–detected tumor response for locally advanced rectal cancer predicts survival outcomes: MERCURY experience.* Journal of Clinical Oncology, 2011. **29**(28): p. 3753-3760.

26. Patel, U.B., et al., *MRI after treatment of locally advanced rectal cancer: how to report tumor response—the MERCURY experience.* American Journal of Roentgenology, 2012. **199**(4): p. W486-W495.

27. Aaronson, N.K., et al., *The European Organization for Research and Treatment of Cancer QLQ-C30: a quality-of-life instrument for use in international clinical trials in oncology.* J Natl Cancer Inst, 1993. **85**(5): p. 365-76.

28. Gujral, S., et al., *Assessing quality of life in patients with colorectal cancer: an update of the EORTC quality of life questionnaire.* European journal of cancer, 2007. **43**(10): p. 1564-1573.

For peer review only

29. Emmertsen, K.J. and S. Laurberg, *Low anterior resection syndrome score: development and validation of a symptom-based scoring system for bowel dysfunction after low anterior resection for rectal cancer.* Annals of surgery, 2012. **255**(5): p. 922-928.
30. Calvert, M., et al., *Reporting of patient-reported outcomes in randomized trials: the CONSORT PRO extension.* Jama, 2013. **309**(8): p. 814-822.
31. Fayers, P., et al., *EORTC QLQ–C30 scoring manual*. 1995: European Organisation for Research and Treatment of Cancer.
32. DAMOCLES Study Group, *A proposed charter for clinical trial data monitoring committees: helping them to do their job well.* The Lancet, 2005. **365**(9460): p. 711-722.
33. MRC Clinical Trials Unit, *Template IDMC Charter version 2.01.* 2006.
34. Soekhai, V., et al., *Discrete choice experiments in health economics: past, present and future.* Pharmacoeconomics, 2019. **37**(2): p. 201-226.
35. Ryan, M., *Discrete choice experiments in health care*. 2004, British Medical Journal Publishing Group.
36. Lancsar, E., D.G. Fiebig, and A.R. Hole, *Discrete choice experiments: a guide to model specification, estimation and software.* Pharmacoeconomics, 2017. **35**(7): p. 697-716.
37. Coast, J., et al., *Using qualitative methods for attribute development for discrete choice experiments: issues and recommendations.* Health economics, 2012. **21**(6): p. 730-741.
38. Couwenberg, A.M., et al., *Utility scores and preferences for surgical and organ-sparing approaches for treatment of intermediate and high-risk rectal cancer.* Diseases of the Colon & Rectum, 2018. **61**(8): p. 911-919.
39. Gani, C., et al., *Organ preservation in rectal cancer: The patients' perspective.* Frontiers in oncology, 2019. **9**: p. 318.

For peer review only

Multi-disciplinary team deems patient unsuitable for radical TME resection of their tumour either because they are considered to be at increased surgical risk or they would find it difficult to manage a stoma.

Eligibility criteria

Biopsy proven non-metastatic rectal adenocarcinoma; ECOG PS 0-2
Primary tumour T1-T3b (T3b: 1-5mm spread beyond muscularis propria)
N0 or Nx on pelvic MRI; Maximum tumour diameter 4cm
Maximum height of superior aspect of tumour is the S2-3 junction

Randomise 1:2 (104 patients total)

Standard dose CRT 50.4 Gy in 28 fractions to mesorectal volume treating once daily Mon-Fri over 5.5 weeks using IMRT, with concurrent fluoropyrimidine chemotherapy*

Escalated dose CRT 50.4 Gy to mesorectal volume, with boost to total 62 Gy to tumour, in 28 fractions treating once daily Mon-Fri over 5.5 weeks using synchronous integrated boost IMRT, with concurrent fluoropyriidine chemotherapy*

Follow-up Schedule (post-start of CRT)

7.5 weeks (2 weeks post CRT completion): Phone follow-up for toxicity (CTCAE v5.0) and Performance Status (PS)
3m: Clinical exam, PS and digital rectal exam (DRE), flexible sigmoidoscopy, MRI pelvis, toxicity, PROM (EORTC C30, EORTC CR29, LARS score)
6m: Clinical exam, PS, DRE, flexible sigmoidoscopy, MRI pelvis, toxicity, PROM
9m: Clinical exam, PS, DRE, toxicity, PROM
12m: Clinical exam, PS, DRE, flexible sigmoidoscopy, toxicity, PROM
24m: Clinical exam, PS, DRE toxicity, PROM

Primary end point: Clinical complete response at 6 months

Secondary end points: Acute toxicity, treatment compliance, late toxicity, patient reported outcome measures (PROMs) including health-related quality of life (HRQoL), colostomy rate, overall survival

*At treating team discretion, concurrent chemotherapy can be used either at 75% dose or omitted completely. This choice must be declared prior to randomisation

For peer review only - http://bmjopen.bmj.com/site/about/guidelines.xhtml

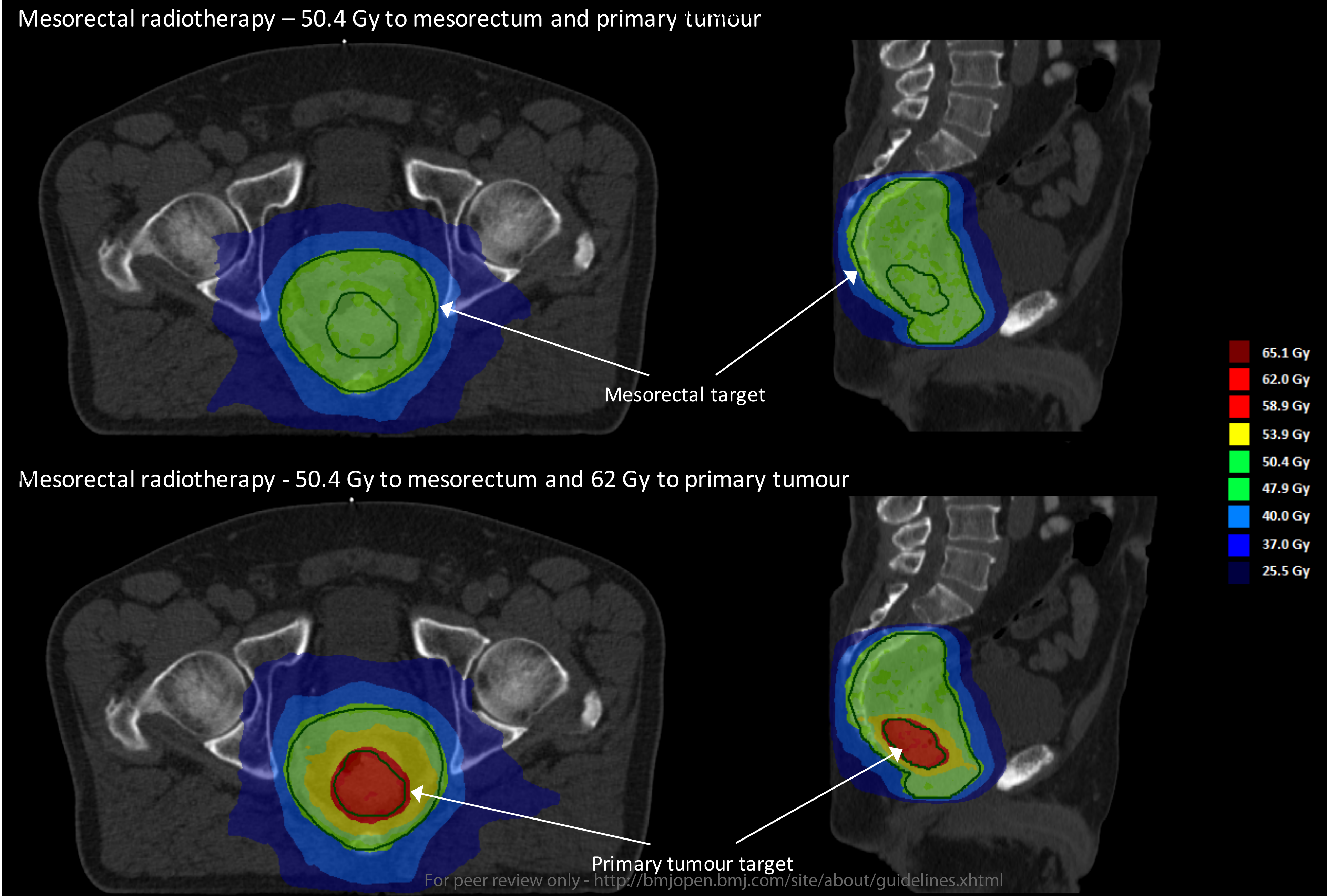

For peer review only - http://bmjopen.bmj.com/site/about/guidelines.xhtml

SPIRIT

STANDARD PROTOCOL ITEMS: RECOMMENDATIONS FOR INTERVENTIONAL TRIALS

SPIRIT 2013 Checklist: Recommended items to address in a clinical trial protocol and related documents*

Section/item	Item No	Description	Addressed on page number
**Administrative information**			
Title	1	Descriptive title identifying the study design, population, interventions, and, if applicable, trial acronym	1
Trial registration	2a	Trial identifier and registry name. If not yet registered, name of intended registry	4, 19
	2b	All items from the World Health Organization Trial Registration Data Set	Yes
Protocol version	3	Date and version identifier	19
Funding	4	Sources and types of financial, material, and other support	22
Roles and responsibilities	5a	Names, affiliations, and roles of protocol contributors	1, 22
	5b	Name and contact information for the trial sponsor	22
	5c	Role of study sponsor and funders, if any, in study design; collection, management, analysis, and interpretation of data; writing of the report; and the decision to submit the report for publication, including whether they will have ultimate authority over any of these activities	22
	5d	Composition, roles, and responsibilities of the coordinating centre, steering committee, endpoint adjudication committee, data management team, and other individuals or groups overseeing the trial, if applicable (see Item 21a for data monitoring committee)	16, 17

For peer review only - http://bmjopen.bmj.com/site/about/guidelines.xhtml

**Introduction**			
Background and rationale	6a	Description of research question and justification for undertaking the trial, including summary of relevant studies (published and unpublished) examining benefits and harms for each intervention	6,7,8_________
	6b	Explanation for choice of comparators	13__________
Objectives	7	Specific objectives or hypotheses	9,10 ________
Trial design	8	Description of trial design including type of trial (e.g., parallel group, crossover, factorial, single group), allocation ratio, and framework (e.g., superiority, equivalence, non-inferiority, exploratory)	8,9 _________
**Methods: Participants, interventions, and outcomes**			
Study setting	9	Description of study settings (e.g., community clinic, academic hospital) and list of countries where data will be collected. Reference to where list of study sites can be obtained	8,9_______
Eligibility criteria	10	Inclusion and exclusion criteria for participants. If applicable, eligibility criteria for study centres and individuals who will perform the interventions (e.g., surgeons, psychotherapists)	11, Table 1 __
Interventions	11a	Interventions for each group with sufficient detail to allow replication, including how and when they will be administered	13_______
	11b	Criteria for discontinuing or modifying allocated interventions for a given trial participant (e.g., drug dose change in response to harms, participant request, or improving/worsening disease)	________
	11c	Strategies to improve adherence to intervention protocols, and any procedures for monitoring adherence (e.g., drug tablet return, laboratory tests)	17 _______
	11d	Relevant concomitant care and interventions that are permitted or prohibited during the trial	11 _______
Outcomes	12	Primary, secondary, and other outcomes, including the specific measurement variable (e.g., systolic blood pressure), analysis metric (e.g., change from baseline, final value, time to event), method of aggregation (e.g., median, proportion), and time point for each outcome. Explanation of the clinical relevance of chosen efficacy and harm outcomes is strongly recommended	9, 10 ____
Participant timeline	13	Time schedule of enrolment, interventions (including any run-ins and washouts), assessments, and visits for participants. A schematic diagram is highly recommended (see Figure)	Table 2, Figure 1

For peer review only - http://bmjopen.bmj.com/site/about/guidelines.xhtml

Sample size	14	Estimated number of participants needed to achieve study objectives and how it was determined, including clinical and statistical assumptions supporting any sample size calculations	12, 13_______
Recruitment	15	Strategies for achieving adequate participant enrolment to reach target sample size	12 ______

Methods: Assignment of interventions (for controlled trials)

Allocation:

Sequence generation	16a	Method of generating the allocation sequence (e.g., computer-generated random numbers), and list of any factors for stratification. To reduce predictability of a random sequence, details of any planned restriction (eg, blocking) should be provided in a separate document that is unavailable to those who enrol participants or assign interventions	12 __________
Allocation concealment mechanism	16b	Mechanism of implementing the allocation sequence (e.g., central telephone; sequentially numbered, opaque, sealed envelopes), describing any steps to conceal the sequence until interventions are assigned	12 ___________
Implementation	16c	Who will generate the allocation sequence, who will enrol participants, and who will assign participants to interventions	12 _________
Blinding (masking)	17a	Who will be blinded after assignment to interventions (e.g., trial participants, care providers, outcome assessors, data analysts), and how	N/A _________
	17b	If blinded, circumstances under which unblinding is permissible, and procedure for revealing a participant's allocated intervention during the trial	N/A__________

Methods: Data collection, management, and analysis

Data collection methods	18a	Plans for assessment and collection of outcome, baseline, and other trial data, including any related processes to promote data quality (e.g., duplicate measurements, training of assessors) and a description of study instruments (e.g., questionnaires, laboratory tests) along with their reliability and validity, if known. Reference to where data collection forms can be found, if not in the protocol	14, (Figure 1), (Table 2)
	18b	Plans to promote participant retention and complete follow-up, including list of any outcome data to be collected for participants who discontinue or deviate from intervention protocols	15 __________

Data management	19	Plans for data entry, coding, security, and storage, including any related processes to promote data quality (e.g., double data entry; range checks for data values). Reference to where details of data management procedures can be found, if not in the protocol	16, 17 _________
Statistical methods	20a	Statistical methods for analysing primary and secondary outcomes. Reference to where other details of the statistical analysis plan can be found, if not in the protocol	15,16______
	20b	Methods for any additional analyses (e.g., subgroup and adjusted analyses)	15, 16_______
	20c	Definition of analysis population relating to protocol non-adherence (e.g., as randomised analysis), and any statistical methods to handle missing data (e.g., multiple imputation)	15, 16 ______
**Methods: Monitoring**			
Data monitoring	21a	Composition of data monitoring committee (DMC); summary of its role and reporting structure; statement of whether it is independent from the sponsor and competing interests; and reference to where further details about its charter can be found, if not in the protocol. Alternatively, an explanation of why a DMC is not needed	17_____
	21b	Description of any interim analyses and stopping guidelines, including who will have access to these interim results and make the final decision to terminate the trial	15 ________
Harms	22	Plans for collecting, assessing, reporting, and managing solicited and spontaneously reported adverse events and other unintended effects of trial interventions or trial conduct	14, 16 ____
Auditing	23	Frequency and procedures for auditing trial conduct, if any, and whether the process will be independent from investigators and the sponsor	_________
**Ethics and dissemination**			
Research ethics approval	24	Plans for seeking research ethics committee/institutional review board (REC/IRB) approval	19 __________
Protocol amendments	25	Plans for communicating important protocol modifications (e.g., changes to eligibility criteria, outcomes, analyses) to relevant parties (e.g., investigators, REC/IRBs, trial participants, trial registries, journals, regulators)	____________

For peer review only - http://bmjopen.bmj.com/site/about/guidelines.xhtml

Consent or assent	26a	Who will obtain informed consent or assent from potential trial participants or authorised surrogates, and how (see Item 32)	12 ___________
	26b	Additional consent provisions for collection and use of participant data and biological specimens in ancillary studies, if applicable	12, 18________
Confidentiality	27	How personal information about potential and enrolled participants will be collected, shared, and maintained in order to protect confidentiality before, during, and after the trial	17 ________
Declaration of interests	28	Financial and other competing interests for principal investigators for the overall trial and each study site	22 ___________
Access to data	29	Statement of who will have access to the final trial dataset, and disclosure of contractual agreements that limit such access for investigators	17 ________
Ancillary and post-trial care	30	Provisions, if any, for ancillary and post-trial care, and for compensation to those who suffer harm from trial participation	N/A ________
Dissemination policy	31a	Plans for investigators and sponsor to communicate trial results to participants, healthcare professionals, the public, and other relevant groups (e.g., via publication, reporting in results databases, or other data sharing arrangements), including any publication restrictions	19 _____
	31b	Authorship eligibility guidelines and any intended use of professional writers	22 __________
	31c	Plans, if any, for granting public access to the full protocol, participant-level dataset, and statistical code	17 _________
**Appendices**			
Informed consent materials	32	Model consent form and other related documentation given to participants and authorised surrogates	_________
Biological specimens	33	Plans for collection, laboratory evaluation, and storage of biological specimens for genetic or molecular analysis in the current trial and for future use in ancillary studies, if applicable	18__________

*It is strongly recommended that this checklist be read in conjunction with the SPIRIT 2013 Explanation & Elaboration for important clarification on the items. Amendments to the protocol should be tracked and dated. The SPIRIT checklist is copyrighted by the SPIRIT Group under the Creative Commons "Attribution-NonCommercial-NoDerivs 3.0 Unported" license.

For peer review only - http://bmjopen.bmj.com/site/about/guidelines.xhtml

BMJ Open

A Phase II trial of Higher RadiOtherapy Dose In The Eradication of early rectal cancer (APHRODITE): Protocol for a multicentre open label randomised controlled trial

Journal:	*BMJ Open*
Manuscript ID	bmjopen-2021-049119.R1
Article Type:	Protocol
Date Submitted by the Author:	20-Sep-2021
Complete List of Authors:	Hudson, Eleanor; University of Leeds, Leeds Institute of Clinical Trials Research<br>Noutch, Samantha; University of Leeds, Leeds Institute of Clinical Trials Research<br>Brown, Sarah; University of Leeds, Leeds Institute of Clinical Trials Research<br>Adapala, Ravi; Wrexham Maelor Hospital, Department of Radiology<br>Bach, Simon; Queen Elizabeth Hospital Birmingham, Academic Department of Surgery<br>Burnett, Carole ; St James's University Hospital, Leeds Cancer Centre<br>Burrage, Alwyn; Patient advocate<br>Gilbert, Alexandra; University of Leeds, Leeds Institute of Medical Research at St James's<br>Hawkins, Maria; University College London, Medical Physics and Biomedical Engineering<br>Howard, Debra; Mount Vernon Cancer Centre, National Radiotherapy Trials QA (RTTQA) Group<br>Jefford, Monica; Patient advocate<br>Kochhar, Rohit; The Christie NHS Foundation Trust<br>Saunders, Mark ; The Christie NHS Foundation Trust<br>Seligmann, Jenny; University of Leeds, Leeds Institute of Medical Research at St James's<br>Smith, Alexandra; University of Leeds, Leeds Institute of Clinical Trials Research<br>Teo, Mark; St James's University Hospital, Leeds Cancer Centre<br>Webb, Edward; University of Leeds, Leeds Institute of Health Sciences<br>Webster, Amanda; Mount Vernon Hospital, National Radiotherapy Trials QA (RTTQA) Group<br>West, Nicholas; University of Leeds, Leeds Institute of Medical Research at St James's<br>Sebag-Montefiore, David ; University of Leeds, Leeds Institute of Medical Research at St James's<br>Gollins, Simon; Glan Clwyd Hospital, North Wales Cancer Treatment Centre<br>Appelt, Ane; University of Leeds, Leeds Institute of Medical Research at St James's
**Primary Subject Heading**:	Oncology

Secondary Subject Heading:	Oncology
Keywords:	Clinical trials < THERAPEUTICS, Gastrointestinal tumours < ONCOLOGY, RADIOTHERAPY

SCHOLARONE™ Manuscripts

A Phase II trial of Higher RadiOtherapy Dose In The Eradication of early rectal cancer (APHRODITE): Protocol for a multicentre open label randomised controlled trial

Authors:

Eleanor M Hudson[1], Samantha Noutch[1], Sarah Brown[1], Ravi Adapala[2], Simon P Bach[3], Carole Burnett[4], Alwyn Burrage[5], Alexandra Gilbert[6], Maria Hawkins[7], Debra Howard[8], Monica Jefford[5], Rohit Kochhar[9], Mark Saunders[9], Jenny Seligmann[6], Alexandra Smith[1], Mark Teo[4], Edward JD Webb[10], Amanda Webster[8], Nicholas West[6], David Sebag-Montefiore[6], Simon Gollins[11]*, Ane Appelt[6]*

*These authors contributed equally and share last authorship

Author affiliation:

[1]Clinical Trials Research Unit, Leeds Institute of Clinical Trials Research, University of Leeds, Leeds, United Kingdom

[2]Department of Radiology, Wrexham Maelor Hospital, Wrexham, United Kingdom

[3]Academic Department of Surgery, Queen Elizabeth Hospital Birmingham, United Kingdom

[4]Leeds Cancer Centre, St James's University Hospital, Leeds, United Kingdom

[5]Patient Advocate, United Kingdom

[6]Leeds Institute of Medical Research at St James's, University of Leeds, Leeds, United Kingdom

[7]Medical Physics and Biomedical Engineering, University College London, London, United Kingdom

[8]National Radiotherapy Trials QA (RTTQA) Group, Mount Vernon Cancer Centre, Middlesex, United Kingdom

[9]The Christie NHS Foundation Trust, Manchester, United Kingdom

[10]Academic Unit of Health Economics, Leeds Institute of Health Sciences, University of Leeds, Leeds, United Kingdom

[11]North Wales Cancer Treatment Centre, Glan Clwyd Hospital, Bodelwyddan, United Kingdom

For peer review only

Corresponding author: Dr. Ane Appelt
A.L.Appelt@leeds.ac.uk
Leeds Institute of Medical Research at St James's,
University of Leeds,
St James's University Hospital,
Harehills
Leeds,
LS9 7LP
+44 113 206 7685 (office)

Word count (excluding title page, abstract, references, figures and tables): 3882

For peer review only

ABSTRACT

Introduction

The standard of care for patients with localised rectal cancer is radical surgery; often combined with pre-operative neoadjuvant (chemo-)radiotherapy. While oncologically effective, this treatment strategy is associated with operative mortality risks, significant morbidity, and stoma formation. An alternative approach is chemoradiotherapy to try to achieve a sustained clinical complete response (cCR). This non-surgical management can be attractive, particularly for patients at high risk of surgical complications. Modern radiotherapy techniques allow increased treatment conformality, enabling increased radiation dose to the tumour while reducing dose to normal tissue. The objective of this trial is to assess if radiotherapy dose escalation increases the cCR rate, with acceptable toxicity, for treatment of early rectal cancer patients unsuitable for radical surgery.

Methods and analysis

APHRODITE is a multicentre open label, randomised controlled phase II trial aiming to recruit 104 participants from 10-12 UK sites. Participants will be allocated with a 2:1 ratio of intervention:control. The intervention is escalated dose radiotherapy (62 Gy to primary tumour, 50.4 Gy to surrounding mesorectum in 28 fractions) using synchronous integrated boost (SIB). The control arm will receive 50.4 Gy to the primary tumour and surrounding mesorectum. Both arms will utilise intensity modulated radiotherapy (IMRT) and daily image guidance, combined with concurrent chemotherapy (Capecitabine, 5-Fluorouracil / Leucovorin or omitted). The primary endpoint is the proportion of participants with cCR at 6-months post start of treatment. Secondary outcomes include early and late toxicities, time to stoma formation, overall survival, and patient reported outcomes (QLQ-C30, QLQ-CR29, LARS questionnaire).

For peer review only

Conclusion

The APHRODITE study will investigate the efficacy and safety of escalated dose radiotherapy for treatment of early stage rectal cancer patients unsuitable for surgery. The results will inform future trials and clinical care.

Trial registration

ISRCTN: 16158514. Date of registry 17th October 2019.

Funding

Yorkshire Cancer Research (YCR).

For peer review only

STRENGTHS AND WEAKNESSES

- APHRODITE is a prospective, multi-centre randomised controlled trial, in contrast to previous studies of organ preservation that have primarily been retrospective, single centre or single arm.
- The trial offers treatment for patients who are not good candidates for (standard) surgical management, thus serving a group of patients often excluded from clinical trials.
- Long course radiotherapy may be intolerable for some very frail patients, for whom short course treatment may be preferential; this study will not assess short course radiotherapy.
- The phase II design is focused on generating preliminary evidence of efficacy; a subsequent phase III trial will be required to evaluate longer-term clinical impact.
- The study has embedded a strong focus on patients and their assessment of outcomes, including patient reported outcome measures (PROMs) and a translational sub-study of patient treatment preferences.

For peer review only

INTRODUCTION

Colorectal cancer is the third most common cancer in the United Kingdom (UK). Each year around 42,000 new cases are diagnosed, with over one quarter within the rectum [1]. The standard of care for patients with localised rectal cancer is radical surgery combined with a selective use of pre-operative radiotherapy and adjuvant chemotherapy. Radical surgery consists of total mesorectal excision (TME) which is an oncologically effective treatment for early stage rectal cancer; only 2% and 12% of patients will experience local or distant failure respectively [2-5]. However, radical surgery is associated with risks of operative mortality, significant morbidity, and the frequent need for stoma.

Mortality following elective major colorectal cancer surgery has fallen significantly in recent years and the 2020 UK National Bowel Cancer Audit Annual Report, relating to 2018-2019, shows overall 90 day mortality of 3% [6]. However, post-operative mortality for rectal cancer increases with age as demonstrated in the Dutch TME trial [7, 8] and is highly dependent on patients' general physical fitness, with significantly increased 30-day mortality in patients with American Society of Anaesthesiologists (ASA) grade III and above physical status [9].

In the 2020 UK National Bowel Cancer Audit [6], 86% of rectal cancer patients had a stoma following major resection, including 78% of patients undergoing anterior resection. Eighteen months later 29% of these patients still had a stoma. Stoma-related morbidity affects at least 50% of patients; with the most common problems including high-output stomas, stoma prolapses, small bowel obstruction and wound infections [10]. Amongst the patients not undergoing radical resection, many are older or frailer, with comorbidities, or those that may struggle to manage a stoma. Surgery is often not a good option for these patients yet there is no standard alternative treatment.

For peer review only

An alternative approach to surgery is the use of radiotherapy with or without chemotherapy (CRT) to try to achieve a sustained clinical complete response (cCR) and potential long-term tumour control. Registration studies of patients with (primarily) locally advanced cancer who have achieved a cCR after neoadjuvant treatment, and opted for non-surgical management, have reported on the oncological safety of this approach [11-13]. A systematic review of 23 cohort studies of patients managed non-surgically following cCR suggested a pooled 2-year local regrowth rate of 21.3% in 575 cCR patients. The vast majority (93.2%) of patients with local regrowth were subsequently managed with salvage surgery [14]. Consequently, a policy of 'active surveillance' is increasingly being adopted, for patients in whom post-CRT endoscopy and magnetic resonance imaging (MRI) suggest a cCR.

Most published studies have focused on an 'opportunistic' approach in patients achieving a cCR following CRT as part of standard clinical management rather than exploring dedicated organ preservation strategies (planned intent). Only a small minority of locally advanced rectal cancers achieve a cCR with standard CRT, however, changing our focus to earlier cancer, which are more likely to respond to chemoradiotherapy, there is the potential for considerably improving response rates. This opens for studies of dedicated and upfront non-surgical management strategies, using chemoradiotherapy as a definitive treatment.

There is moderate evidence of a radiation dose-response relationship for pathological tumour regression after preoperative (chemo-)radiotherapy [15, 16]. Currently, there is no high-level evidence as to whether dose escalation increases the cCR rate, although selected publications have reported high cCR and organ preservation rates with high-dose chemoradiotherapy for early cancers [17, 18]. Early reponse and toxicity data for a single-arm phase II organ preservation study delivering 62Gy (tumour) and 50.4Gy (lymph nodes) have also been presented in abstract form [19]. The non-randomised nature of these studies is a major limitation, particularly with the inclusion of small and early cancers which are more likely to respond [20]. Additionally, there is limited evidence of the optimum dose level for dose escalation, though experience from anal

For peer review only

cancer and preliminary data from two ongoing Danish studies [NCT02438839, NCT04095299] indicate that 62 Gy can be safely delivered to a confined target volume in 2.2Gy per fraction.

Modern radiotherapy techniques, including Intensity Modulated Radiotherapy (IMRT) and volumetric arc therapy (VMAT) with image guidance (IGRT), improve conformality of treated volumes. Theoretically this will reduce early and late toxicity when compared to conventional 3D conformal planning [21]. These advances offer the possibility of increasing the dose to the tumour without excessive morbidity of surrounding normal tissues. Dose escalation with simultaneous integrated boost (SIB) can thus be achieved without significantly altering the dose delivered to the organs at risk, and without extending the overall treatment time.

To further minimise radiation-induced side effects, a risk-adapted target volume can be used. The risk of pelvic lymph node involvement or distal mesorectal nodal involvement is very low for patients with early rectal cancer [22, 23]. Additionally, local recurrences are unlikely to be found above the level of the S2/3 interspace [24]. In early rectal cancer, it is thus doubtful that traditional, large elective treatment volumes are indicated. Therefore, it is reasonable to reduce the target volume to the peritumoral region of the primary tumour and the mesorectum [25, 26].

The APHRODITE phase II study will utilise modern radiotherapy techniques to investigate the efficacy and safety of escalated dose radiotherapy for non-surgical treatment of early stage rectal cancer in patients not suitable for standard surgical management.

METHODS AND ANALYSIS

Design and aim

APHRODITE is a phase II, multi-centre, open-label, randomised-controlled trial of IMRT, with select use of concomitant chemotherapy, in patients with early stage rectal cancer (T1-3bN0,

For peer review only

maximum diameter ≤ 4 cm) who are deemed not suitable for radical TME surgery by their multidisciplinary team (MDT). The primary aim is to assess whether radiotherapy dose escalation increases the cCR rate at 6 months from the start of CRT, compared with standard radiotherapy dose CRT, with acceptable toxicity. Patients may receive full dose, reduced dose or no concominant chemotherapy. A total of 104 eligible patients will be recruited from 10-12 UK radiotherapy sites. Participants will be randomised on a 2:1 basis to receive escalated dose or standard dose chemoradiation. The participant pathway can be seen schematically in Figure 1. The study protocol and this manuscript have been written in accordance with Standard Protocol Items: Recommendations for Interventional Trials (SPIRIT) guidelines.

Trial objectives

The primary objective of APHRODITE is to compare the proportion of participants with a cCR at 6-months from the start of CRT. A composite definition for cCR is used, including digital rectal examination (DRE), rectal endoscopy and pelvic MRI, defined by:

1. No evidence of either mucosal tumour or submucosal swelling on white light endoscopy. A flat white scar remains, with or without telangiectasia.
2. No palpable tumour upon DRE.
3. High resolution pelvic MRI scanning shows either a linear scar only, or dense fibrosis with no obvious tumour signal (mrTRG 1 or 2) [27, 28].

This definition of cCR follows international consensus [29]. Every effort will be made to ensure trimodality assessment (endoscopy, DRE and MRI). If such is not possible, confirmation should always include a rectal endoscopy and one other (DRE, MRI). Detailed guidance on MRI and endoscopy is provided in separate trial guidelines. The protocol does not require use of biopsy for response evaluation.

Currently research is unclear whether high dose radiotherapy increases the rate of superficial mucosal ulcerations in the presence of a complete primary tumour response. Therefore, an

For peer review only

alternative definition of the primary endpoint, with the inclusion of superficial mucosal ulceration classified as cCR, will be explored in a sensitivity analysis.

The secondary objectives of APHRODITE evaluate the safety and tolerability of dose escalation based on:

- Acute toxicities as per Common Terminology Criteria for Adverse Events (CTCAE V.5.0.) (measured weekly during and up to two weeks post end of treatment).
- Treatment compliance (both radiotherapy and, if received, concomitant chemotherapy).
- Toxicities during follow-up as per CTCAE V5.0 (measured up to the 24-month follow-up point).

The longer-term activity of dose escalation will be evaluated based on:

- Time to stoma formation (time from randomisation to stoma formation – colostomy or ileostomy).
- Overall survival (time from randomisation to death from any cause).
- Patient reported outcome measures (PROMs), assessed by the European Organisation for Research and Treatment of Cancer (EORTC) QLQ-C30 [30], EORTC QLQ-CR29 [31], Low Anterior Resection Syndrome (LARS) questionnaire [32] and additional EORTC-QLQ items from the Item Library relevant for organ preservation.

Study population

Eligible patients are those with early stage rectal cancer who are deemed by their MDT not suitable for radical TME surgery because either:

i. The patient is thought to be at increased surgical risk due to specific medical co-morbidity or general frailty.
ii. The patient has marked anxiety at the prospect of having a stoma.
iii. It is anticipated that the patient would have difficulty managing a stoma post-operatively e.g. due to physical problems.

For peer review only

230 See Table 1 for a full list of inclusion and exclusion criteria.

For peer review only

Table 1– Inclusion and exclusion criteria

Inclusion criteria	Exclusion criteria
1. Biopsy confirmed adenocarcinoma of the rectum	1. Nodal involvement on MRI (i.e. N1- N2) * or discontinuous tumour deposits (N1c)
2. Age 18 years or over	2. The presence of EMVI discontinuous with the primary tumour on MRI
3. Able to provide written informed consent	3. Involvement of anal intersphincteric plane or external anal sphincter or adjacent organs; or tumour involves/breaches levator ani
4. Patient deemed unsuitable for TME surgical resection, see main text for details	4. Tumour grown through and breached mesorectal fascia
5. Patient suitable for pelvic radiotherapy or chemoradiation: ECOG PS 0-2	5. Signet ring carcinoma or tumours histopathologically containing a neuroendocrine component; or dominant mucinous tumour on MRI
6. Primary tumour staged T1-T3b *, and maximum tumour diameter ≤ 4 cm, both on MRI	6. Undergone an attempt at complete local resection of their cancer
7. No unequivocally involved lymph nodes, NX and N0 eligible *	7. Definite distant metastases (equivocal distant metastases are permitted)
8. Tumour visible on MRI	8. Defunctioning colostomy/ileostomy fashioned
9. Superior aspect of tumour is at or below a horizontal line drawn from the anterior aspect of the S2/3 junction on pre-treatment MRI	9. Previous pelvic radiotherapy; or prior systemic chemotherapy for colorectal cancer
10. For low rectal tumours, superior to the puborectalis sling, the mesorectal fascia or levator is:<br>○ Clear (>1mm from disease to levator ani or mesorectal fascia)<br>○ Or threatened (≤1mm from disease to levator ani or mesorectal fascia)<br>○ Or mesorectal fascia is involved but not breached	10. Prior invasive malignancy unless disease free for a minimum of 3 years (excluding basal cell carcinoma of the skin or other in situ carcinomas)
11. Blood counts fulfilling:<br>○ Estimated creatinine clearance ≥ 50 ml/min<br>○ Absolute neutrophil count > 1.5 x 109/l; platelets > 100 x 109/l<br>○ Serum transaminase concentration < 3 x Upper Limit Normal (ULN)<br>Bilirubin concentration < 1.5 x ULN	11. Women who are pregnant, breastfeeding or of child bearing potential and unwilling to use contraceptives

** TNM staging as per UICC 8th Edition. EMVI = extramural venous invasion.*

For peer review only

Randomisation and recruitment

A computer-generated minimisation program incorporating a random element will be used to allocate patients on a 2:1 basis to receive escalated dose or standard dose chemoradiation, ensuring arms are well-balanced for the following factors:

- Randomising site
- T-stage (<T3 vs. T3)
- Chemotherapy use (either 100% or 75% dose) vs. no chemotherapy use

Randomisation will be performed centrally via the University of Leeds Clinical Trials Research Unit (CTRU) automated 24-hour randomisation system. The registration and randomisation process will be instigated by on-site research staff; patient consent form must be attained prior to registration.

Recruitment will take place over 2 years, with an estimated monthly recruitment rate of 4-6 patients per month. APHRODITE opened in February 2020, with expected recruitment completion by winter 2022. The study has and will be presented at national and international meetings, increasing clinical awareness. A trial website and dedicated twitter account will be utilised to further increase exposure, highlighting the trial population criteria to treating clinicians and MDT members. Based on preliminary feedback from clinicians and patient representatives in the study design phase, the 2:1 allocation between the intervention and control arms will make recruitment attractive for patients with limited standard treatment options.

Sample size

The target sample size is 104 participants (70 experimental: 34 control), based on a two-group chi-squared test of equal proportions, with continuity correction. Assuming a control arm cCR rate of 35% [33], using a one-sided test at 20% significance, 104 participants (incorporating a 5% loss to follow-up) will provide 80% power to detect an absolute difference of 20% (to 55%) between

For peer review only

arms. Data suggests potential differences as great as 25% (in favour of the experimental arm) [17, 34, 35].

An inflated type I error rate of 20% is used as the aim is to show preliminary evidence of activity [36, 37]. The direct comparison with the control avoids the risk of incorrect decision making due to uncertain historical control data [38].

Treatment regimen

Intervention arm: the primary tumour will receive 62 Gy in 28 fractions, with 50.4Gy delivered to the surrounding elective mesorectal volume, using a simultaneously integrated boost.

Control arm: the primary tumour and elective mesorectal volume will receive a uniform 50.4Gy in 28 fractions (pre-operation standard dose).

A reduced mesorectum-only elective volume will be used, as appropriate for early stage disease at low risk of nodal failure. All treatment will be planned and delivered using IMRT or VMAT, with extensive radiotherapy guidelines, based on the STAR-TREC trial target definition and treatment planning [25, 26]. Daily image guidance with cone beam CT will be mandatory and promotes precise treatment delivery; essential as the mesorectum and tumour exhibit significant changes in day-to-day position [7, 24]. See Figure 2 for an example treatment plan.

Concurrent chemotherapy will be used during CRT, either as single agent oral capecitabine (825 mg/m^2) given twice per day Monday-Friday on the days of radiotherapy throughout the course of radiotherapy, or alternatively as intravenous 5-Fluorouracil/Leucovorin delivered once per day (5FU 350mg/m^2 plus Leucovorin) concurrent with fractions 1-5 and 20-25 of radiotherapy (weeks 1 and 5). If the treating clinician decides a patient is not fit to receive the full (100%) dose of chemotherapy, due to co-morbidities, or general frailty, they have the options to treat at 75% dose, or omit chemotherapy (radiotherapy alone). The intended chemotherapy treatment option must be declared for each patient *prior* to randomisation.

For peer review only

Trial assessments and follow-up

All willing patients will be assessed prior to randomisation to confirm full eligibility (Table 1). Recruited participants will be assessed at various timepoints (Table 2).

Table 2 – Trial schedule of assessment

	Early assessments			Treatment		Follow-up assessment (measured post CRT)					
	Eligibility	Baseline	Pre-treat.	On treat.	End of treat.	2w	3m	6 m	9 m	12 m	24 m
Informed consent	X										
Histopathology	X										
Performance status	X		X		X	X	X	X	X	X	X
Bloods	X		X	X	X						
Pregnancy screening	X										
ECG		X									
MRI (pelvis)	X						X	X	Local schedule		
CT (chest, abdo, pelvis)	X						Local standard schedule				
Flexible sigmoidoscopy	X						X	X		X	
Digital rectal examination	X						X	X	X	X	X
Clinical assessment	X			X	X		X	X	X	X	X
Toxicity (CTCAE)		X		X	X	X	X	X	X	X	X
PROMs		X			X		X	X	X	X	X

For further detail of the participation flow see trial schema (Figure 1).

Adverse Reaction (ARs), Serious Adverse Reactions (SARs), Related Unexpected Serious Adverse Events (RUSAEs) and Serious Adverse Events of Interest (SAEoI) will be collected from randomisation until the 24-month follow-up point. SAEoI are events identified by the research team that need additional monitoring; including angina/myocardial infarction, pulmonary embolism, serious skin reactions.

For peer review only

Data will be collected from all randomised participants, irrespective of treatment compliance. Where possible, endpoint and PROMs data will be collected from withdrawn participants who do not withdraw consent from further data collection.

Treatment and response assessment quality assurance

The trial will implement robust quality assurance of radiotherapy treatment and the primary endpoint assessments. The radiotherapy quality assurance (QA) programme will be implemented by the National Radiotherapy Trials Quality Assurance Group to ensure treatment is planned and delivered according to the trial protocol. All radiotherapy centres will complete pre-treatment benchmark exercises. Anonymised pre-treatment, 3-month and 6-month endoscopic photographs and MRI scans will be collected for all patients, with the first 2 patients from each site reviewed centrally. Trial Management Group (TMG) specialist members will ensure the inclusion/exclusion criteria have been met (baseline scan) and response has been correctly reported (3 and 6 months), providing feedback where necessary. Further review may be performed in batches.

Statistical analysis

A full statistical analysis plan will be written before any analyses are undertaken. No formal interim analyses are planned within the trial.

The primary endpoint will be assessed on an Modified *Intention to treat (MITT) population*; all randomised participants that have had atleast one dose of trial treatment, grouped by the treatment arm to which they were randomised (regardless of ineligibility, non-compliance or withdrawal) [39].

A multivariable logistic regression model, adjusted for the minimisation factors, will be used to compare the proportion of participants achieving a cCR at 6-months. Significance testing will be 1-sided at the 20% level. The treatment effect will be presented as an odds ratio with a two-sided

For peer review only

60% confidence interval (equivalent to a 1-sided 80% lower confidence limit). Patterns of missing data and imputation methods (including multiple imputation) will be explored, if appropriate. A sensitivity analysis will be performed using the alternative definition of cCR.

All secondary analyses will use a 5% significance level and a two-sided confidence interval, where appropriate. Safety data will be presented on the *safety population*; participants are summarised on the treatment received regardless of randomisation. All other data will be summarised on an MITT population.

Summary statistics will be presented for toxicities on the number and proportion of participants experiencing ARs, SARs, RUSAEs, and SAEoI. Treatment compliance to the allocated radiotherapy (+/- chemotherapy) will be monitored and presented, including summaries for delays, dose modifications and discontinuation. PROMs will be summarised and reported using standard guidelines [32, 40, 41]. Mixed effects models will be used to assess differences between treatment arms. Cumulative incidence will be used to compare the time to stoma formation between treatment arms. Overall survival will be compared using the Cox Proportional Hazards model. Kaplan-Meier curves and survival estimates with corresponding confidence intervals will also be presented.

Trial organisation

Trial coordination, data management and statistical analysis will be directed and conducted by the trial specific project team at the University of Leeds CTRU. Trial supervision will be established according to the principles of Good Clinical Practice and in line with the relevant Research Governance Framework within the UK and CTRU standard operating procedures.

For peer review only

Data collection and management

Participating sites will record participant data on trial-specific case report forms and submit them to the CTRU. Participants will complete PROMs on the relevant paper questionnaire forms at the time of their clinic appointment. All trial data will be entered, validated and stored securely by CTRU. Missing and discrepant data will be flagged and additional data validations raised, as appropriate, from the CTRU data management team. Missing data (except PROMs) will be followed-up until received, or confirmed as not available. Data items regarding consent, patient safety and the primary endpoint will be subject to manual priority checking.

Data will be stored securely at Leeds CTRU. Only CTRU will have access to the data, prior to analysis and release of trial results. Upon completion of the trial, data will be stored in the sponsor archiving facility for a minimum of 15 years. After the final trial results have been published, interested researchers may contact the TMG and CTRU to request access to relevant data. Any requests will be reviewed by the TMG.

Trial monitoring

The TMG will provide ongoing clinical, practical and statistical advice on trial-related matters. The trial will be overseen by an independent Trial Steering Committee (TSC) and Data Monitoring and Ethics Committee (DMEC). The DMEC comprises two clinical oncologists and one statistician. They will review and monitor accumulating interim safety data and unblinded reports, at least annually. Their role is to protect the safety of the participants and maintain the research integrity of the study, advising the TSC on trial developments, including advice on trial continuation. A trial-specific DMEC charter has been developed in line with DAMOCLES [42, 43]. The sponsor and TSC have ultimate oversight over the conduct and continuation of the trial.

Patient and Public Involvement

For peer review only

APHRODITE has been developed with patient and public involvement (PPI) representatives from the earliest stages. Multiple aspects of both trial design and delivery have been shaped in collaboration with our PPI representatives, including the acceptability of the intervention, randomisation and the allocation ratio (2:1). One PPI representative was a co-applicant on the grant application (MJ). Two PPI representatives are members of the TMG (MJ, AB), and have provided patient perspective throughout set-up and recruitment. Upon their suggestion, APHRODITE has a patient-facing trial website, and a patient newsletter will be issued at regular intervals, with updates of trial progress and results when available. Trial feedback and communication with participants has been highlighted as a key importance by our PPI representatives.

Patient preference Sub-study

Dose escalation is associated with a greater probability of tumour control at the cost of greater risk of toxicity, yet little information exists as to what risks patients are willing to accept for a better chance of tumour control. The APHRODITE patient preference study (Chief Investigator, Dr Edward Webb), to be conducted alongside the main trial, aims to address this knowledge gap.

The patient preference sub-study uses a well-established survey technique called a discrete choice experiment (DCE) [44]. DCEs have a simple response format in which patients make a series of choices between hypothetical treatments characterised by attributes, such as probability of tumour control after two years and the risk of side effects [45]. The attribute levels change in each question, allowing the trade-offs participants make to be analysed [46]. The survey has been developed using qualitative methods in line with good practice recommendations [47]. Analysis of responses will quantify maximum acceptable risk of toxicity for a given probability of tumour control, as well as their preferences for process attributes, such as the number of treatment sessions and after-treatment support. Participants will complete the survey before treatment and six months after treatment. This will allow us to assess whether preferences are systematically influenced by treatment experience.

For peer review only

The sub-study plans to recruit 100-200 participants from sites participating in the APHRODITE trial, including APHRODITE participants, patients who decline APHRODITE participation, in addition to patients who are being managed non-surgically but do not meet the trial inclusion/exclusion criteria. Separate ethical approval has been obtained for the patient preference study from an NHS research ethics committee (reference 19/NE/0249).

Sample collection

Pre-treatment routine diagnostic formalin fixed paraffin embedded tumour biopsy tissue samples will be collected on all patients and sent to the University of Leeds, for storage. Consent will be taken for their use in future translational research. The routine glass Haematoxylin & Eosin stained slides used for diagnosis will be scanned to create a digital pathology resource for QA of the trial and support translational research, including research examining biomarkers of radiosensitivity and radioresistance.

ETHICS AND DISSEMINATION

The trial obtained ethical approval from North West Greater Manchester East research ethic committee (reference no. 19/NW/0565) and is registered in the ISRCTN registry (registration number 16158514). Confirmed by the UK Medicines and Healthcare products Regulation Agency, APHRODITE is not a trial of an investigational medicinal product (non-CTIMP). The trial is currently adhering to protocol version 2.0 (approved 31/03/2020).

The primary endpoint and early toxicity data will be analysed and reported when the last participant reaches 6-months post start of treatment. Further analyses will be conducted when all patients have reached 24-months follow-up. The final trial publication will be written by the TMG, and will adhere to ICMJE guidelines.

For peer review only

DISCUSSION

There is currently a lack of prospective trials of dedicated non-surgical management strategies in rectal cancer. It is therefore unclear whether increased radiotherapy dose may contribute to short-term tumour response, long-term local control and organ preservation. In particular, the lack of randomised data from the early phase setting has made it difficult to elucidate whether the high response rates observed in phase II trials are due to patient selection or treatment. The APHRODITE trial will provide high-level evidence whether dose escalation increases the cCR rate when directly compared to standard dose. Further studies will be required to provide evidence for whether a higher radiotherapy dose can provide improved long-term local control without surgery.

Other ongoing studies are examining dedicated organ preservation strategies for early rectal cancer. The STAR-TReC phase III trial (ISRCTN14240288) randomises patients with early (T1-3bN0) cancer with a preference for organ preservation between short- and long-course standard (chemo-)radiotherapy, with the aim of non-surgical management or local excision only. Both trials remain complimentary: STAR TREC uses standard radiotherapy in a patient population suitable for surgery, while APHRODITE will assess intensified treatment in an alternative patient group. Three other studies of radiotherapy dose escalation strategies for organ preservation are ongoing: The international OPERA phase III trial (NCT02505750) randomises patients between moderately dose escalated CRT (54Gy) and standard dose CRT (45Gy) followed by Papillon contact X-ray therapy (90Gy in 3 fractions); the single-centre randomised phase II MORPHEUS trial (NCT03051464) randomises patients between moderately dose escalated CRT (54Gy) and CRT (45Gy) followed by brachytherapy (30Gy in 3 fractions); and the Danish multi-centre WW3 phase II trial (NCT04095299) randomises patients between dose-escalated CRT (62Gy) and standard dose CRT (50.4Gy). All these studies require suitability for surgery, and the first two depend on

For peer review only

access to specialised treatment equipment. The APHRODITE study thus fills a clear research gap in the current international trial portfolio.

Studies of non-surgical management and organ preservation for rectal cancer have focused on clinical endpoints, e.g. cCR rate, stoma rate, and overall survival, as well as the clinicians' interpretation of patient outcome. There is a paucity of toxicity data including PROMs following nonsurgical management, particularly in a patient population usually excluded from clinical trials. The APHRODITE study will prospectively measure toxicity and PROMs, providing a valuable and unique dataset.

At present, there is a lack of understanding of how patients weigh and prioritise different potential outcomes, including the balance between efficacy and toxicity, in the setting of non-surgical rectal cancer treatment. The decision regarding which severity of treatment-related toxicities is acceptable for a given organ preservation strategy has previously been evaluated purely by clinicians. Two small studies examined patient preferences for organ-preserving and surgical treatment approaches [48, 49]. However, no studies have focused specifically on patient attitudes to treatment characteristics and outcomes following non-surgical management. The APHRODITE patient preference sub-study will help inform future dose escalation studies and help non-surgical management of rectal cancer to better reflect patient perspectives.

CONCLUSION

The APHRODITE trial will provide valuable, high quality, patient-centred evidence for escalated dose radiotherapy in rectal cancer patients not suitable for radical surgery, addressing an important research gap.

For peer review only

AUTHOR CONTRIBUTIONS

Contributors to the conception and design: AA, DSM, SB, SG.

Development of the protocol, trial documents and patient information sheet: AA, AB, AG, AS, AW, CB, DSM, DH, EH, JS, MJ, MS, NW, RA, RK, SB, SG, SN.

Writing manuscript: EH, AA.

Reviewed manuscript: AB, AG, AS, AW, CB, DSM, EW, MH, MS, MT, NW, SB, SPB, SG.

All authors read and approved the final manuscript.

FUNDING, SPONSOR AND COORDINATION

APHRODITE is supported by Yorkshire Cancer Research (award number L411). The study is coordinated by Leeds CTRU and sponsored by University of Leeds (GA18/118305). For enquires trial contact *email: APHRODITE@leeds.ac.uk.* Trial website: https://ctru.leeds.ac.uk/aphrodite/.

Both the sponsor and funder have no contribution to the study design and concept; collection, management, analysis, and interpretation of data; the writing of and decision to submit reports for publication. The sponsor and funder will review all manuscripts prior to publication.

COMPETING INTERESTS

EW reports grants from Yorkshire Cancer Research, during the conduct of the study.

MS reports personal fees from Servier, personal fees from Amgen, personal fees from Marck, outside the submitted work.

NW reports grants from Yorkshire Cancer Research, during the conduct of the study; grants from Cancer Research UK, outside the submitted work.

For peer review only

EXCLUSIVE LICENCE

I, the Submitting Author has the right to grant and does grant on behalf of all authors of the Work (as defined in the below author licence), an exclusive licence and/or a non-exclusive licence for contributions from authors who are: i) UK Crown employees; ii) where BMJ has agreed a CC-BY licence shall apply, and/or iii) in accordance with the terms applicable for US Federal Government officers or employees acting as part of their official duties; on a worldwide, perpetual, irrevocable, royalty-free basis to BMJ Publishing Group Ltd ("BMJ") its licensees and where the relevant Journal is co-owned by BMJ to the co-owners of the Journal, to publish the Work in BMJ Open and any other BMJ products and to exploit all rights, as set out in our licence.

The Submitting Author accepts and understands that any supply made under these terms is made by BMJ to the Submitting Author unless you are acting as an employee on behalf of your employer or a postgraduate student of an affiliated institution which is paying any applicable article publishing charge ("APC") for Open Access articles. Where the Submitting Author wishes to make the Work available on an Open Access basis (and intends to pay the relevant APC), the terms of reuse of such Open Access shall be governed by a Creative Commons licence – details of these licences and which Creative Commons licence will apply to this Work are set out in our licence referred to above.

FIGURE LEGEND: Figure 1 - *Trial Schema*

FIGURE LEGEND: Figure 2 - *Example treatment plans for the control arm (top panels, uniform 50.4 Gy in 28 fractions to the primary tumour and elective mesorectal volume); and intervention arm (lower panels, 62 Gy in 28 fractions to the primary tumour, 50.4Gy to the elective mesorectal volume), for a single patient.*

For peer review only

REFERENCES

1. CRUK. *Bowel cancer statistics*. 2017 [cited 2020; Available from: https://www.cancerresearchuk.org/health-professional/cancer-statistics/incidence.

2. Bentrem, D.J., et al., *T1 adenocarcinoma of the rectum: transanal excision or radical surgery?* Annals of surgery, 2005. **242**(4): p. 472.

3. Endreseth, B.H., et al., *Transanal excision vs. major surgery for T1 rectal cancer.* Diseases of the colon & rectum, 2005. **48**(7): p. 1380-1388.

4. Peeters, K.C., et al., *The TME trial after a median follow-up of 6 years: increased local control but no survival benefit in irradiated patients with resectable rectal carcinoma.* Annals of surgery, 2007. **246**(5): p. 693-701.

5. Sebag-Montefiore, D., et al., *Preoperative radiotherapy versus selective postoperative chemoradiotherapy in patients with rectal cancer (MRC CR07 and NCIC-CTG C016): a multicentre, randomised trial.* The Lancet, 2009. **373**(9666): p. 811-820.

6. *National Bowel Cancer Audit Annual Report 2020*. 2020.

7. Kapiteijn, E., et al., *Preoperative Radiotherapy Combined with Total Mesorectal Excision for Resectable Rectal Cancer.* New England Journal of Medicine, 2001. **345**(9): p. 638-646.

8. Tekkis, P.P., et al., *Operative mortality in colorectal cancer: prospective national study.* Bmj, 2003. **327**(7425): p. 1196-1201.

9. Rutten, H.J., et al., *Controversies of total mesorectal excision for rectal cancer in elderly patients.* The lancet oncology, 2008. **9**(5): p. 494-501.

10. Bakx, R., et al., *Morbidity of temporary loop ileostomies.* Digestive surgery, 2004. **21**(4): p. 277-281.

11. Habr-Gama, A., et al., *Complete clinical response after neoadjuvant chemoradiation therapy for distal rectal cancer: characterization of clinical and endoscopic findings for standardization.* Diseases of the Colon & Rectum, 2010. **53**(12): p. 1692-1698.

For peer review only

12. Van Der Valk, M.J., et al., *Long-term outcomes of clinical complete responders after neoadjuvant treatment for rectal cancer in the International Watch & Wait Database (IWWD): an international multicentre registry study.* The Lancet, 2018. **391**(10139): p. 2537-2545.
13. Renehan, A.G., et al., *Watch-and-wait approach versus surgical resection after chemoradiotherapy for patients with rectal cancer (the OnCoRe project): a propensity-score matched cohort analysis.* The Lancet Oncology, 2016. **17**(2): p. 174-183.
14. Sammour, T., et al., *Nonoperative management or 'watch and wait'for rectal cancer with complete clinical response after neoadjuvant chemoradiotherapy: a critical appraisal.* Annals of surgical oncology, 2017. **24**(7): p. 1904-1915.
15. Appelt, A.L., et al., *Radiation dose-response model for locally advanced rectal cancer after preoperative chemoradiation therapy.* International Journal of Radiation Oncology* Biology* Physics, 2013. **85**(1): p. 74-80.
16. Valentini, V., et al., *The INTERACT Trial: Long-term results of a randomised trial on preoperative capecitabine-based radiochemotherapy intensified by concomitant boost or oxaliplatin, for cT2 (distal)–cT3 rectal cancer.* Radiotherapy and Oncology, 2019. **134**: p. 110-118.
17. Appelt, A.L., et al., *High-dose chemoradiotherapy and watchful waiting for distal rectal cancer: a prospective observational study.* The Lancet oncology, 2015. **16**(8): p. 919-927.
18. Dizdarevic, E., et al., *Long-term patient-reported outcomes after high-dose chemoradiation therapy for nonsurgical management of distal rectal cancer.* International Journal of Radiation Oncology* Biology* Physics, 2020. **106**(3): p. 556-563.
19. Jensen, L.H., et al., *400MO Curative chemoradiation for low rectal cancer: Early clinical outcomes from a multicentre phase II trial.* Annals of Oncology, 2020. **31**(Suppl. 4): p. S411.

20. Maas, M., et al., *Long-term outcome in patients with a pathological complete response after chemoradiation for rectal cancer: a pooled analysis of individual patient data.* The lancet oncology, 2010. **11**(9): p. 835-844.

21. Appelt, A.L. and D. Sebag-Montefiore, *Technological advances in radiotherapy of rectal cancer: opportunities and challenges.* Current opinion in oncology, 2016. **28**(4): p. 353-358.

22. Roels, S., et al., *Definition and delineation of the clinical target volume for rectal cancer.* International Journal of Radiation Oncology* Biology* Physics, 2006. **65**(4): p. 1129-1142.

23. Leibold, T., et al., *Prognostic implications of the distribution of lymph node metastases in rectal cancer after neoadjuvant chemoradiotherapy.* Journal of clinical oncology, 2008. **26**(13): p. 2106-2111.

24. Nijkamp, J., et al., *Three-dimensional analysis of recurrence patterns in rectal cancer: the cranial border in hypofractionated preoperative radiotherapy can be lowered.* International Journal of Radiation Oncology* Biology* Physics, 2011. **80**(1): p. 103-110.

25. Appelt, A.L., et al., *Robust dose planning objectives for mesorectal radiotherapy of early stage rectal cancer–A multicentre dose planning study.* Technical Innovations & Patient Support in Radiation Oncology, 2019. **11**: p. 14-21.

26. Peters, F.P., et al., *Mesorectal radiotherapy for early stage rectal cancer: A novel target volume.* Clinical and Translational Radiation Oncology, 2020. **21**: p. 104-111.

27. Patel, U.B., et al., *Magnetic resonance imaging–detected tumor response for locally advanced rectal cancer predicts survival outcomes: MERCURY experience.* Journal of Clinical Oncology, 2011. **29**(28): p. 3753-3760.

28. Patel, U.B., et al., *MRI after treatment of locally advanced rectal cancer: how to report tumor response—the MERCURY experience.* American Journal of Roentgenology, 2012. **199**(4): p. W486-W495.

For peer review only

29. Fokas, E., et al., *International consensus recommendations on key outcome measures for organ preservation after (chemo) radiotherapy in patients with rectal cancer.* Nature Reviews Clinical Oncology, 2021: p. 1-12.
30. Aaronson, N.K., et al., *The European Organization for Research and Treatment of Cancer QLQ-C30: a quality-of-life instrument for use in international clinical trials in oncology.* J Natl Cancer Inst, 1993. **85**(5): p. 365-76.
31. Gujral, S., et al., *Assessing quality of life in patients with colorectal cancer: an update of the EORTC quality of life questionnaire.* European journal of cancer, 2007. **43**(10): p. 1564-1573.
32. Emmertsen, K.J. and S. Laurberg, *Low anterior resection syndrome score: development and validation of a symptom-based scoring system for bowel dysfunction after low anterior resection for rectal cancer.* Annals of surgery, 2012. **255**(5): p. 922-928.
33. Dossa, F., et al., *A watch-and-wait approach for locally advanced rectal cancer after a clinical complete response following neoadjuvant chemoradiation: a systematic review and meta-analysis.* The Lancet Gastroenterology hepatology, 2017. **2**(7): p. 501-513.
34. Appelt, A.L., et al., *Radiation dose-response model for locally advanced rectal cancer after preoperative chemoradiation therapy.* International Journal of Radiation Oncology Biology Physics, 2013. **85**(1): p. 74-80.
35. Appelt, A.L. and A. Jakobsen, *Radiation techniques for increasing local control in the non-surgical management of rectal cancer.* Current Colorectal Cancer Reports, 2015. **11**(5): p. 267-274.
36. Rubinstein, L.V., et al., *Design issues of randomized phase II trials and a proposal for phase II screening trials.* Journal of Clinical Oncology, 2005. **23**(28): p. 7199-7206.
37. Korn, E.L., et al., *Design issues in randomized phase II/III trials.* Journal of Clinical Oncology, 2012. **30**(6): p. 667.

For peer review only

38. Ratain, M.J. and D. Sargent, *Optimising the design of phase II oncology trials: the importance of randomisation.* European journal of cancer, 2009. **45**(2): p. 275-280.

39. Abraha, I. and A. Montedori, *Modified intention to treat reporting in randomised controlled trials: systematic review.* Bmj, 2010. **340**.

40. Calvert, M., et al., *Reporting of patient-reported outcomes in randomized trials: the CONSORT PRO extension.* Jama, 2013. **309**(8): p. 814-822.

41. Fayers, P., et al., *EORTC QLQ–C30 scoring manual.* 1995: European Organisation for Research and Treatment of Cancer.

42. DAMOCLES Study Group, *A proposed charter for clinical trial data monitoring committees: helping them to do their job well.* The Lancet, 2005. **365**(9460): p. 711-722.

43. MRC Clinical Trials Unit, *Template IDMC Charter version 2.01.* 2006.

44. Soekhai, V., et al., *Discrete choice experiments in health economics: past, present and future.* Pharmacoeconomics, 2019. **37**(2): p. 201-226.

45. Ryan, M., *Discrete choice experiments in health care*. 2004, British Medical Journal Publishing Group.

46. Lancsar, E., D.G. Fiebig, and A.R. Hole, *Discrete choice experiments: a guide to model specification, estimation and software.* Pharmacoeconomics, 2017. **35**(7): p. 697-716.

47. Coast, J., et al., *Using qualitative methods for attribute development for discrete choice experiments: issues and recommendations.* Health economics, 2012. **21**(6): p. 730-741.

48. Couwenberg, A.M., et al., *Utility scores and preferences for surgical and organ-sparing approaches for treatment of intermediate and high-risk rectal cancer.* Diseases of the Colon & Rectum, 2018. **61**(8): p. 911-919.

49. Gani, C., et al., *Organ preservation in rectal cancer: The patients' perspective.* Frontiers in oncology, 2019. **9**: p. 318.

For peer review only

Multi-disciplinary team deems patient unsuitable for radical TME resection of their tumour either because they are considered to be at increased surgical risk or they would find it difficult to manage a stoma.

Eligibility criteria

Biopsy proven non-metastatic rectal adenocarcinoma; ECOG PS 0-2
Primary tumour T1-T3b (T3b: 1-5mm spread beyond muscularis propria)
N0 or Nx on pelvic MRI; Maximum tumour diameter 4cm
Maximum height of superior aspect of tumour is the S2-3 junction

Randomise 1:2 (104 patients total)

Standard dose CRT 50.4 Gy in 28 fractions to mesorectal volume treating once daily Mon-Fri over 5.5 weeks using IMRT, with concurrent fluoropyrimidine chemotherapy*

Escalated dose CRT 50.4 Gy to mesorectal volume, with boost to total 62 Gy to tumour, in 28 fractions treating once daily Mon-Fri over 5.5 weeks using synchronous integrated boost IMRT, with concurrent fluoropyriidine chemotherapy*

Follow-up Schedule (post-start of CRT)

7.5 weeks (2 weeks post CRT completion): Phone follow-up for toxicity (CTCAE v5.0) and Performance Status (PS)
3m: Clinical exam, PS and digital rectal exam (DRE), flexible sigmoidoscopy, MRI pelvis, toxicity, PROM (EORTC C30, EORTC CR29, LARS score)
6m: Clinical exam, PS, DRE, flexible sigmoidoscopy, MRI pelvis, toxicity, PROM
9m: Clinical exam, PS, DRE, toxicity, PROM
12m: Clinical exam, PS, DRE, flexible sigmoidoscopy, toxicity, PROM
24m: Clinical exam, PS, DRE toxicity, PROM

Primary end point: Clinical complete response at 6 months

Secondary end points: Acute toxicity, treatment compliance, late toxicity, patient reported outcome measures (PROMs) including health-related quality of life (HRQoL), colostomy rate, overall survival

*At treating team discretion, concurrent chemotherapy can be used either at 75% dose or omitted completely. This choice must be declared prior to randomisation

For peer review only - http://bmjopen.bmj.com/site/about/guidelines.xhtml

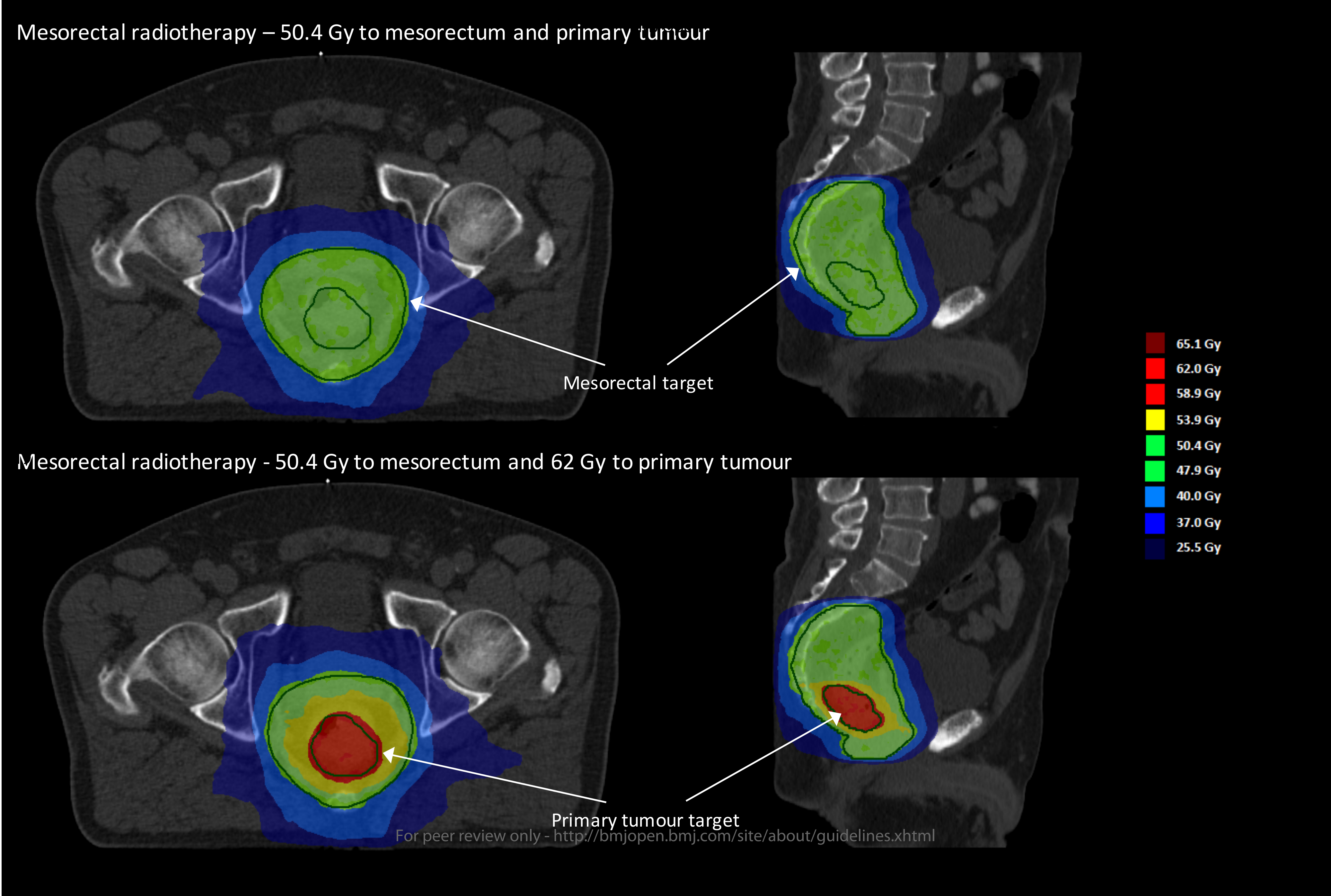

For peer review only - http://bmjopen.bmj.com/site/about/guidelines.xhtml

SPIRIT
STANDARD PROTOCOL ITEMS: RECOMMENDATIONS FOR INTERVENTIONAL TRIALS

SPIRIT 2013 Checklist: Recommended items to address in a clinical trial protocol and related documents*

Section/item	Item No	Description	Addressed on page number
**Administrative information**			
Title	1	Descriptive title identifying the study design, population, interventions, and, if applicable, trial acronym	1 ___________
Trial registration	2a	Trial identifier and registry name. If not yet registered, name of intended registry	4, 19__________
	2b	All items from the World Health Organization Trial Registration Data Set	Yes __________
Protocol version	3	Date and version identifier	20 ___________
Funding	4	Sources and types of financial, material, and other support	23 ___________
Roles and responsibilities	5a	Names, affiliations, and roles of protocol contributors	1, 23 _________
	5b	Name and contact information for the trial sponsor	23 ___________
	5c	Role of study sponsor and funders, if any, in study design; collection, management, analysis, and interpretation of data; writing of the report; and the decision to submit the report for publication, including whether they will have ultimate authority over any of these activities	23 ___________
	5d	Composition, roles, and responsibilities of the coordinating centre, steering committee, endpoint adjudication committee, data management team, and other individuals or groups overseeing the trial, if applicable (see Item 21a for data monitoring committee)	17, 18 ________

For peer review only

**Introduction**			
Background and rationale	6a	Description of research question and justification for undertaking the trial, including summary of relevant studies (published and unpublished) examining benefits and harms for each intervention	6,7,8_________
	6b	Explanation for choice of comparators	14__________
Objectives	7	Specific objectives or hypotheses	9,10 ________
Trial design	8	Description of trial design including type of trial (e.g., parallel group, crossover, factorial, single group), allocation ratio, and framework (e.g., superiority, equivalence, non-inferiority, exploratory)	8,9 _________
**Methods: Participants, interventions, and outcomes**			
Study setting	9	Description of study settings (e.g., community clinic, academic hospital) and list of countries where data will be collected. Reference to where list of study sites can be obtained	8,9_______
Eligibility criteria	10	Inclusion and exclusion criteria for participants. If applicable, eligibility criteria for study centres and individuals who will perform the interventions (e.g., surgeons, psychotherapists)	12, Table 1 __
Interventions	11a	Interventions for each group with sufficient detail to allow replication, including how and when they will be administered	14_______
	11b	Criteria for discontinuing or modifying allocated interventions for a given trial participant (e.g., drug dose change in response to harms, participant request, or improving/worsening disease)	________
	11c	Strategies to improve adherence to intervention protocols, and any procedures for monitoring adherence (e.g., drug tablet return, laboratory tests)	18 _______
	11d	Relevant concomitant care and interventions that are permitted or prohibited during the trial	12 _______
Outcomes	12	Primary, secondary, and other outcomes, including the specific measurement variable (e.g., systolic blood pressure), analysis metric (e.g., change from baseline, final value, time to event), method of aggregation (e.g., median, proportion), and time point for each outcome. Explanation of the clinical relevance of chosen efficacy and harm outcomes is strongly recommended	9, 10 ____
Participant timeline	13	Time schedule of enrolment, interventions (including any run-ins and washouts), assessments, and visits for participants. A schematic diagram is highly recommended (see Figure)	Table 2, Figure 1

For peer review only - http://bmjopen.bmj.com/site/about/guidelines.xhtml

Sample size	14	Estimated number of participants needed to achieve study objectives and how it was determined, including clinical and statistical assumptions supporting any sample size calculations	13, 14_______
Recruitment	15	Strategies for achieving adequate participant enrolment to reach target sample size	13 ______

Methods: Assignment of interventions (for controlled trials)

Allocation:

Sequence generation	16a	Method of generating the allocation sequence (e.g., computer-generated random numbers), and list of any factors for stratification. To reduce predictability of a random sequence, details of any planned restriction (eg, blocking) should be provided in a separate document that is unavailable to those who enrol participants or assign interventions	13 __________
Allocation concealment mechanism	16b	Mechanism of implementing the allocation sequence (e.g., central telephone; sequentially numbered, opaque, sealed envelopes), describing any steps to conceal the sequence until interventions are assigned	13 ___________
Implementation	16c	Who will generate the allocation sequence, who will enrol participants, and who will assign participants to interventions	13 _________
Blinding (masking)	17a	Who will be blinded after assignment to interventions (e.g., trial participants, care providers, outcome assessors, data analysts), and how	N/A _________
	17b	If blinded, circumstances under which unblinding is permissible, and procedure for revealing a participant's allocated intervention during the trial	N/A__________

Methods: Data collection, management, and analysis

Data collection methods	18a	Plans for assessment and collection of outcome, baseline, and other trial data, including any related processes to promote data quality (e.g., duplicate measurements, training of assessors) and a description of study instruments (e.g., questionnaires, laboratory tests) along with their reliability and validity, if known. Reference to where data collection forms can be found, if not in the protocol	15, (Figure 1), (Table 2)
	18b	Plans to promote participant retention and complete follow-up, including list of any outcome data to be collected for participants who discontinue or deviate from intervention protocols	16, 18________

Data management	19	Plans for data entry, coding, security, and storage, including any related processes to promote data quality (e.g., double data entry; range checks for data values). Reference to where details of data management procedures can be found, if not in the protocol	18 _________
Statistical methods	20a	Statistical methods for analysing primary and secondary outcomes. Reference to where other details of the statistical analysis plan can be found, if not in the protocol	16,17______
	20b	Methods for any additional analyses (e.g., subgroup and adjusted analyses)	16, 17_______
	20c	Definition of analysis population relating to protocol non-adherence (e.g., as randomised analysis), and any statistical methods to handle missing data (e.g., multiple imputation)	16, 17 ______
**Methods: Monitoring**			
Data monitoring	21a	Composition of data monitoring committee (DMC); summary of its role and reporting structure; statement of whether it is independent from the sponsor and competing interests; and reference to where further details about its charter can be found, if not in the protocol. Alternatively, an explanation of why a DMC is not needed	18_____
	21b	Description of any interim analyses and stopping guidelines, including who will have access to these interim results and make the final decision to terminate the trial	16 ________
Harms	22	Plans for collecting, assessing, reporting, and managing solicited and spontaneously reported adverse events and other unintended effects of trial interventions or trial conduct	15, 17 ____
Auditing	23	Frequency and procedures for auditing trial conduct, if any, and whether the process will be independent from investigators and the sponsor	_________
**Ethics and dissemination**			
Research ethics approval	24	Plans for seeking research ethics committee/institutional review board (REC/IRB) approval	20 __________
Protocol amendments	25	Plans for communicating important protocol modifications (e.g., changes to eligibility criteria, outcomes, analyses) to relevant parties (e.g., investigators, REC/IRBs, trial participants, trial registries, journals, regulators)	____________

For peer review only - http://bmjopen.bmj.com/site/about/guidelines.xhtml

Consent or assent	26a	Who will obtain informed consent or assent from potential trial participants or authorised surrogates, and how (see Item 32)	13 ___________
	26b	Additional consent provisions for collection and use of participant data and biological specimens in ancillary studies, if applicable	13, 20________
Confidentiality	27	How personal information about potential and enrolled participants will be collected, shared, and maintained in order to protect confidentiality before, during, and after the trial	18 ________
Declaration of interests	28	Financial and other competing interests for principal investigators for the overall trial and each study site	23 ___________
Access to data	29	Statement of who will have access to the final trial dataset, and disclosure of contractual agreements that limit such access for investigators	18 ________
Ancillary and post-trial care	30	Provisions, if any, for ancillary and post-trial care, and for compensation to those who suffer harm from trial participation	N/A ________
Dissemination policy	31a	Plans for investigators and sponsor to communicate trial results to participants, healthcare professionals, the public, and other relevant groups (e.g., via publication, reporting in results databases, or other data sharing arrangements), including any publication restrictions	19 _____
	31b	Authorship eligibility guidelines and any intended use of professional writers	23 __________
	31c	Plans, if any, for granting public access to the full protocol, participant-level dataset, and statistical code	18 _________
**Appendices**			
Informed consent materials	32	Model consent form and other related documentation given to participants and authorised surrogates	_________
Biological specimens	33	Plans for collection, laboratory evaluation, and storage of biological specimens for genetic or molecular analysis in the current trial and for future use in ancillary studies, if applicable	20__________

*It is strongly recommended that this checklist be read in conjunction with the SPIRIT 2013 Explanation & Elaboration for important clarification on the items. Amendments to the protocol should be tracked and dated. The SPIRIT checklist is copyrighted by the SPIRIT Group under the Creative Commons "Attribution-NonCommercial-NoDerivs 3.0 Unported" license.

For peer review only - http://bmjopen.bmj.com/site/about/guidelines.xhtml

BMJ Open

A Phase II trial of Higher RadiOtherapy Dose In The Eradication of early rectal cancer (APHRODITE): Protocol for a multicentre open label randomised controlled trial

Journal:	*BMJ Open*
Manuscript ID	bmjopen-2021-049119.R2
Article Type:	Protocol
Date Submitted by the Author:	16-Feb-2022
Complete List of Authors:	Hudson, Eleanor; University of Leeds, Leeds Institute of Clinical Trials Research<br>Noutch, Samantha; University of Leeds, Leeds Institute of Clinical Trials Research<br>Brown, Sarah; University of Leeds, Leeds Institute of Clinical Trials Research<br>Adapala, Ravi; Wrexham Maelor Hospital, Department of Radiology<br>Bach, Simon; Queen Elizabeth Hospital Birmingham, Academic Department of Surgery<br>Burnett, Carole ; St James's University Hospital, Leeds Cancer Centre<br>Burrage, Alwyn; Patient advocate<br>Gilbert, Alexandra; University of Leeds, Leeds Institute of Medical Research at St James's<br>Hawkins, Maria; University College London, Medical Physics and Biomedical Engineering<br>Howard, Debra; Mount Vernon Cancer Centre, National Radiotherapy Trials QA (RTTQA) Group<br>Jefford, Monica; Patient advocate<br>Kochhar, Rohit; The Christie NHS Foundation Trust<br>Saunders, Mark ; The Christie NHS Foundation Trust<br>Seligmann, Jenny; University of Leeds, Leeds Institute of Medical Research at St James's<br>Smith, Alexandra; University of Leeds, Leeds Institute of Clinical Trials Research<br>Teo, Mark; St James's University Hospital, Leeds Cancer Centre<br>Webb, Edward; University of Leeds, Leeds Institute of Health Sciences<br>Webster, Amanda; Mount Vernon Hospital, National Radiotherapy Trials QA (RTTQA) Group<br>West, Nicholas; University of Leeds, Leeds Institute of Medical Research at St James's<br>Sebag-Montefiore, David ; University of Leeds, Leeds Institute of Medical Research at St James's<br>Gollins, Simon; Glan Clwyd Hospital, North Wales Cancer Treatment Centre<br>Appelt, Ane; University of Leeds, Leeds Institute of Medical Research at St James's
**Primary Subject Heading**:	Oncology

Secondary Subject Heading:	Oncology
Keywords:	Clinical trials < THERAPEUTICS, Gastrointestinal tumours < ONCOLOGY, RADIOTHERAPY

SCHOLARONE™
Manuscripts

A Phase II trial of Higher RadiOtherapy Dose In The Eradication of early rectal cancer (APHRODITE): Protocol for a multicentre open label randomised controlled trial

Authors:

Eleanor M Hudson[1], Samantha Noutch[1], Sarah Brown[1], Ravi Adapala[2], Simon P Bach[3], Carole Burnett[4], Alwyn Burrage[5], Alexandra Gilbert[6], Maria Hawkins[7], Debra Howard[8], Monica Jefford[5], Rohit Kochhar[9], Mark Saunders[9], Jenny Seligmann[6], Alexandra Smith[1], Mark Teo[4], Edward JD Webb[10], Amanda Webster[8], Nicholas West[6], David Sebag-Montefiore[6], Simon Gollins[11]*, Ane Appelt[6]*

**These authors contributed equally and share last authorship*

Author affiliation:

[1]Clinical Trials Research Unit, Leeds Institute of Clinical Trials Research, University of Leeds, Leeds, United Kingdom

[2]Department of Radiology, Wrexham Maelor Hospital, Wrexham, United Kingdom

[3]Academic Department of Surgery, Queen Elizabeth Hospital Birmingham, United Kingdom

[4]Leeds Cancer Centre, St James's University Hospital, Leeds, United Kingdom

[5]Patient Advocate, United Kingdom

[6]Leeds Institute of Medical Research at St James's, University of Leeds, Leeds, United Kingdom

[7]Medical Physics and Biomedical Engineering, University College London, London, United Kingdom

[8]National Radiotherapy Trials QA (RTTQA) Group, Mount Vernon Cancer Centre, Middlesex, United Kingdom

[9]The Christie NHS Foundation Trust, Manchester, United Kingdom

[10]Academic Unit of Health Economics, Leeds Institute of Health Sciences, University of Leeds, Leeds, United Kingdom

[11]North Wales Cancer Treatment Centre, Glan Clwyd Hospital, Bodelwyddan, United Kingdom

For peer review only

Corresponding author: Dr. Ane Appelt

A.L.Appelt@leeds.ac.uk

Leeds Institute of Medical Research at St James's,

University of Leeds,

St James's University Hospital,

Harehills

Leeds,

LS9 7LP

+44 113 206 7685 (office)

Word count (excluding title page, abstract, references, figures and tables): 3882

For peer review only

ABSTRACT

Introduction

The standard of care for patients with localised rectal cancer is radical surgery; often combined with pre-operative neoadjuvant (chemo-)radiotherapy. While oncologically effective, this treatment strategy is associated with operative mortality risks, significant morbidity, and stoma formation. An alternative approach is chemoradiotherapy to try to achieve a sustained clinical complete response (cCR). This non-surgical management can be attractive, particularly for patients at high risk of surgical complications. Modern radiotherapy techniques allow increased treatment conformality, enabling increased radiation dose to the tumour while reducing dose to normal tissue. The objective of this trial is to assess if radiotherapy dose escalation increases the cCR rate, with acceptable toxicity, for treatment of early rectal cancer patients unsuitable for radical surgery.

Methods and analysis

APHRODITE is a multicentre open label, randomised controlled phase II trial aiming to recruit 104 participants from 10-12 UK sites. Participants will be allocated with a 2:1 ratio of intervention:control. The intervention is escalated dose radiotherapy (62 Gy to primary tumour, 50.4 Gy to surrounding mesorectum in 28 fractions) using synchronous integrated boost (SIB). The control arm will receive 50.4 Gy to the primary tumour and surrounding mesorectum. Both arms will utilise intensity modulated radiotherapy (IMRT) and daily image guidance, combined with concurrent chemotherapy (Capecitabine, 5-Fluorouracil / Leucovorin or omitted). The primary endpoint is the proportion of participants with cCR at 6-months post start of treatment. Secondary outcomes include early and late toxicities, time to stoma formation, overall survival, and patient reported outcomes (QLQ-C30, QLQ-CR29, LARS questionnaire).

For peer review only

Ethics and dissemination

The trial obtained ethical approval from North West Greater Manchester East research ethic committee (reference no. 19/NW/0565) and is funded by Yorkshire Cancer Research (YCR). The final trial results will be published in peer-reviewed journals and adhere to ICMJE guidelines. Trial registration number ISRCTN: 16158514. Date of registry 17th October 2019.

For peer review only

STRENGTHS AND WEAKNESSES

- APHRODITE is a prospective, multi-centre randomised controlled trial, in contrast to previous studies of organ preservation that have primarily been retrospective, single centre or single arm.
- The trial offers treatment for patients who are not good candidates for (standard) surgical management, thus serving a group of patients often excluded from clinical trials.
- Long course radiotherapy may be intolerable for some very frail patients, for whom short course treatment may be preferential; this study will not assess short course radiotherapy.
- The phase II design is focused on generating preliminary evidence of efficacy; a subsequent phase III trial will be required to evaluate longer-term clinical impact.
- The study has embedded a strong focus on patients and their assessment of outcomes, including patient reported outcome measures (PROMs) and a translational sub-study of patient treatment preferences.

For peer review only

INTRODUCTION

Colorectal cancer is the third most common cancer in the United Kingdom (UK). Each year around 42,000 new cases are diagnosed, with over one quarter within the rectum [1]. The standard of care for patients with localised rectal cancer is radical surgery combined with a selective use of pre-operative radiotherapy and adjuvant chemotherapy. Radical surgery consists of total mesorectal excision (TME) which is an oncologically effective treatment for early stage rectal cancer; only 2% and 12% of patients will experience local or distant failure respectively [2-5]. However, radical surgery is associated with risks of operative mortality, significant morbidity, and the frequent need for stoma.

Mortality following elective major colorectal cancer surgery has fallen significantly in recent years and the 2020 UK National Bowel Cancer Audit Annual Report, relating to 2018-2019, shows overall 90 day mortality of 3% [6]. However, post-operative mortality for rectal cancer increases with age as demonstrated in the Dutch TME trial [7, 8] and is highly dependent on patients' general physical fitness, with significantly increased 30-day mortality in patients with American Society of Anaesthesiologists (ASA) grade III and above physical status [9].

In the 2020 UK National Bowel Cancer Audit [6], 86% of rectal cancer patients had a stoma following major resection, including 78% of patients undergoing anterior resection. Eighteen months later 29% of these patients still had a stoma. Stoma-related morbidity affects at least 50% of patients; with the most common problems including high-output stomas, stoma prolapses, small bowel obstruction and wound infections [10]. Amongst the patients not undergoing radical resection, many are older or frailer, with comorbidities, or those that may struggle to manage a stoma. Surgery is often not a good option for these patients yet there is no standard alternative treatment.

For peer review only

An alternative approach to surgery is the use of radiotherapy with or without chemotherapy (CRT) to try to achieve a sustained clinical complete response (cCR) and potential long-term tumour control. Registration studies of patients with (primarily) locally advanced cancer who have achieved a cCR after neoadjuvant treatment, and opted for non-surgical management, have reported on the oncological safety of this approach [11-13]. A systematic review of 23 cohort studies of patients managed non-surgically following cCR suggested a pooled 2-year local regrowth rate of 21.3% in 575 cCR patients. The vast majority (93.2%) of patients with local regrowth were subsequently managed with salvage surgery [14]. Consequently, a policy of ‘active surveillance’ is increasingly being adopted, for patients in whom post-CRT endoscopy and magnetic resonance imaging (MRI) suggest a cCR.

Most published studies have focused on an ‘opportunistic’ approach in patients achieving a cCR following CRT as part of standard clinical management rather than exploring dedicated organ preservation strategies (planned intent). Only a small minority of locally advanced rectal cancers achieve a cCR with standard CRT, however, changing our focus to earlier cancer, which are more likely to respond to chemoradiotherapy, there is the potential for considerably improving response rates. This opens for studies of dedicated and upfront non-surgical management strategies, using chemoradiotherapy as a definitive treatment.

There is moderate evidence of a radiation dose-response relationship for pathological tumour regression after preoperative (chemo-)radiotherapy [15, 16]. Currently, there is no high-level evidence as to whether dose escalation increases the cCR rate, although selected publications have reported high cCR and organ preservation rates with high-dose chemoradiotherapy for early cancers [17, 18]. Early reponse and toxicity data for a single-arm phase II organ preservation study delivering 62Gy (tumour) and 50.4Gy (lymph nodes) have also been presented in abstract form [19]. The non-randomised nature of these studies is a major limitation, particularly with the inclusion of small and early cancers which are more likely to respond [20]. Additionally, there is limited evidence of the optimum dose level for dose escalation, though experience from anal

For peer review only

For peer review only - http://bmjopen.bmj.com/site/about/guidelines.xhtml

cancer and preliminary data from two ongoing Danish studies [NCT02438839, NCT04095299] indicate that 62 Gy can be safely delivered to a confined target volume in 2.2Gy per fraction.

Modern radiotherapy techniques, including Intensity Modulated Radiotherapy (IMRT) and volumetric arc therapy (VMAT) with image guidance (IGRT), improve conformality of treated volumes. Theoretically this will reduce early and late toxicity when compared to conventional 3D conformal planning [21]. These advances offer the possibility of increasing the dose to the tumour without excessive morbidity of surrounding normal tissues. Dose escalation with simultaneous integrated boost (SIB) can thus be achieved without significantly altering the dose delivered to the organs at risk, and without extending the overall treatment time.

To further minimise radiation-induced side effects, a risk-adapted target volume can be used. The risk of pelvic lymph node involvement or distal mesorectal nodal involvement is very low for patients with early rectal cancer [22, 23]. Additionally, local recurrences are unlikely to be found above the level of the S2/3 interspace [24]. In early rectal cancer, it is thus doubtful that traditional, large elective treatment volumes are indicated. Therefore, it is reasonable to reduce the target volume to the peritumoral region of the primary tumour and the mesorectum [25, 26].

The APHRODITE phase II study will utilise modern radiotherapy techniques to investigate the efficacy and safety of escalated dose radiotherapy for non-surgical treatment of early stage rectal cancer in patients not suitable for standard surgical management.

METHODS AND ANALYSIS

Design and aim

APHRODITE is a phase II, multi-centre, open-label, randomised-controlled trial of IMRT, with select use of concomitant chemotherapy, in patients with early stage rectal cancer (T1-3bN0,

For peer review only

maximum diameter ≤ 4 cm) who are deemed not suitable for radical TME surgery by their multidisciplinary team (MDT). The primary aim is to assess whether radiotherapy dose escalation increases the cCR rate at 6 months from the start of CRT, compared with standard radiotherapy dose CRT, with acceptable toxicity. Patients may receive full dose, reduced dose or no concominant chemotherapy. A total of 104 eligible patients will be recruited from 10-12 UK radiotherapy sites. Participants will be randomised on a 2:1 basis to receive escalated dose or standard dose chemoradiation. The participant pathway can be seen schematically in Figure 1. The study protocol and this manuscript have been written in accordance with Standard Protocol Items: Recommendations for Interventional Trials (SPIRIT) guidelines.

Trial objectives

The primary objective of APHRODITE is to compare the proportion of participants with a cCR at 6-months from the start of CRT. A composite definition for cCR is used, including digital rectal examination (DRE), rectal endoscopy and pelvic MRI, defined by:

1. No evidence of either mucosal tumour or submucosal swelling on white light endoscopy. A flat white scar remains, with or without telangiectasia.
2. No palpable tumour upon DRE.
3. High resolution pelvic MRI scanning shows either a linear scar only, or dense fibrosis with no obvious tumour signal (mrTRG 1 or 2) [27, 28].

This definition of cCR follows international consensus [29]. Every effort will be made to ensure trimodality assessment (endoscopy, DRE and MRI). If such is not possible, confirmation should always include a rectal endoscopy and one other (DRE, MRI). Detailed guidance on MRI and endoscopy is provided in separate trial guidelines. The protocol does not require use of biopsy for response evaluation.

Currently research is unclear whether high dose radiotherapy increases the rate of superficial mucosal ulcerations in the presence of a complete primary tumour response. Therefore, an

For peer review only

alternative definition of the primary endpoint, with the inclusion of superficial mucosal ulceration classified as cCR, will be explored in a sensitivity analysis.

The secondary objectives of APHRODITE evaluate the safety and tolerability of dose escalation based on:

- Acute toxicities as per Common Terminology Criteria for Adverse Events (CTCAE V.5.0.) (measured weekly during and up to two weeks post end of treatment).
- Treatment compliance (both radiotherapy and, if received, concomitant chemotherapy).
- Toxicities during follow-up as per CTCAE V5.0 (measured up to the 24-month follow-up point).

The longer-term activity of dose escalation will be evaluated based on:

- Time to stoma formation (time from randomisation to stoma formation – colostomy or ileostomy).
- Overall survival (time from randomisation to death from any cause).
- Patient reported outcome measures (PROMs), assessed by the European Organisation for Research and Treatment of Cancer (EORTC) QLQ-C30 [30], EORTC QLQ-CR29 [31], Low Anterior Resection Syndrome (LARS) questionnaire [32] and additional EORTC-QLQ items from the Item Library relevant for organ preservation.

Study population

Eligible patients are those with early stage rectal cancer who are deemed by their MDT not suitable for radical TME surgery because either:

i. The patient is thought to be at increased surgical risk due to specific medical co-morbidity or general frailty.
ii. The patient has marked anxiety at the prospect of having a stoma.
iii. It is anticipated that the patient would have difficulty managing a stoma post-operatively e.g. due to physical problems.

See Table 1 for a full list of inclusion and exclusion criteria.

For peer review only

Table 1– Inclusion and exclusion criteria

Inclusion criteria	Exclusion criteria
1. Biopsy confirmed adenocarcinoma of the rectum	1. Nodal involvement on MRI (i.e. N1- N2) * or discontinuous tumour deposits (N1c)
2. Age 18 years or over	2. The presence of EMVI discontinuous with the primary tumour on MRI
3. Able to provide written informed consent	3. Involvement of anal intersphincteric plane or external anal sphincter or adjacent organs; or tumour involves/breaches levator ani
4. Patient deemed unsuitable for TME surgical resection, see main text for details	4. Tumour grown through and breached mesorectal fascia
5. Patient suitable for pelvic radiotherapy or chemoradiation: ECOG PS 0-2	5. Signet ring carcinoma or tumours histopathologically containing a neuroendocrine component; or dominant mucinous tumour on MRI
6. Primary tumour staged T1-T3b *, and maximum tumour diameter ≤ 4 cm, both on MRI	6. Undergone an attempt at complete local resection of their cancer
7. No unequivocally involved lymph nodes, NX and N0 eligible *	7. Definite distant metastases (equivocal distant metastases are permitted)
8. Tumour visible on MRI	8. Defunctioning colostomy/ileostomy fashioned
9. Superior aspect of tumour is at or below a horizontal line drawn from the anterior aspect of the S2/3 junction on pre-treatment MRI	9. Previous pelvic radiotherapy; or prior systemic chemotherapy for colorectal cancer
10. For low rectal tumours, superior to the puborectalis sling, the mesorectal fascia or levator is:<br>○ Clear (>1mm from disease to levator ani or mesorectal fascia)<br>○ Or threatened (≤1mm from disease to levator ani or mesorectal fascia)<br>○ Or mesorectal fascia is involved but not breached	10. Prior invasive malignancy unless disease free for a minimum of 3 years (excluding basal cell carcinoma of the skin or other in situ carcinomas)
11. Blood counts fulfilling:<br>○ Estimated creatinine clearance ≥ 50 ml/min<br>○ Absolute neutrophil count > 1.5 x 109/l; platelets > 100 x 109/l<br>○ Serum transaminase concentration < 3 x Upper Limit Normal (ULN)<br>Bilirubin concentration < 1.5 x ULN	11. Women who are pregnant, breastfeeding or of child bearing potential and unwilling to use contraceptives

** TNM staging as per UICC 8th Edition. EMVI = extramural venous invasion.*

For peer review only

Randomisation and recruitment

A computer-generated minimisation program incorporating a random element will be used to allocate patients on a 2:1 basis to receive escalated dose or standard dose chemoradiation, ensuring arms are well-balanced for the following factors:

- Randomising site
- T-stage (<T3 vs. T3)
- Chemotherapy use (either 100% or 75% dose) vs. no chemotherapy use

Randomisation will be performed centrally via the University of Leeds Clinical Trials Research Unit (CTRU) automated 24-hour randomisation system. The registration and randomisation process will be instigated by on-site research staff; patient consent form must be attained prior to registration (See Supplementary 1).

Recruitment will take place over 2 years, with an estimated monthly recruitment rate of 4-6 patients per month. APHRODITE opened in February 2020, with expected recruitment completion by winter 2022. The study has and will be presented at national and international meetings, increasing clinical awareness. A trial website and dedicated twitter account will be utilised to further increase exposure, highlighting the trial population criteria to treating clinicians and MDT members. Based on preliminary feedback from clinicians and patient representatives in the study design phase, the 2:1 allocation between the intervention and control arms will make recruitment attractive for patients with limited standard treatment options.

Sample size

The target sample size is 104 participants (70 experimental: 34 control), based on a two-group chi-squared test of equal proportions, with continuity correction. Assuming a control arm cCR rate of 35% [33], using a one-sided test at 20% significance, 104 participants (incorporating a 5% loss to follow-up) will provide 80% power to detect an absolute difference of 20% (to 55%) between

For peer review only

arms. Data suggests potential differences as great as 25% (in favour of the experimental arm) [17, 34, 35].

An inflated type I error rate of 20% is used as the aim is to show preliminary evidence of activity [36, 37]. The direct comparison with the control avoids the risk of incorrect decision making due to uncertain historical control data [38].

Treatment regimen

Intervention arm: the primary tumour will receive 62 Gy in 28 fractions, with 50.4Gy delivered to the surrounding elective mesorectal volume, using a simultaneously integrated boost.

Control arm: the primary tumour and elective mesorectal volume will receive a uniform 50.4Gy in 28 fractions (pre-operation standard dose).

A reduced mesorectum-only elective volume will be used, as appropriate for early stage disease at low risk of nodal failure. All treatment will be planned and delivered using IMRT or VMAT, with extensive radiotherapy guidelines, based on the STAR-TREC trial target definition and treatment planning [25, 26]. Daily image guidance with cone beam CT will be mandatory and promotes precise treatment delivery; essential as the mesorectum and tumour exhibit significant changes in day-to-day position [7, 24]. See Figure 2 for an example treatment plan.

Concurrent chemotherapy will be used during CRT, either as single agent oral capecitabine (825 mg/m^2) given twice per day Monday-Friday on the days of radiotherapy throughout the course of radiotherapy, or alternatively as intravenous 5-Fluorouracil/Leucovorin delivered once per day (5FU 350mg/m^2 plus Leucovorin) concurrent with fractions 1-5 and 20-25 of radiotherapy (weeks 1 and 5). If the treating clinician decides a patient is not fit to receive the full (100%) dose of chemotherapy, due to co-morbidities, or general frailty, they have the options to treat at 75% dose, or omit chemotherapy (radiotherapy alone). The intended chemotherapy treatment option must be declared for each patient *prior* to randomisation.

For peer review only

Trial assessments and follow-up

All willing patients will be assessed prior to randomisation to confirm full eligibility (Table 1). Recruited participants will be assessed at various timepoints (Table 2).

Table 2 – Trial schedule of assessment

	Early assessments			Treatment		Follow-up assessment (measured post CRT)					
	Eligibility	Baseline	Pre-treat.	On treat.	End of treat.	2w	3m	6 m	9 m	12 m	24 m
Informed consent	X										
Histopathology	X										
Performance status	X		X		X	X	X	X	X	X	X
Bloods	X		X	X	X						
Pregnancy screening	X										
ECG		X									
MRI (pelvis)	X						X	X	Local schedule		
CT (chest, abdo, pelvis)	X						Local standard schedule				
Flexible sigmoidoscopy	X						X	X		X	
Digital rectal examination	X						X	X	X	X	X
Clinical assessment	X			X	X		X	X	X	X	X
Toxicity (CTCAE)		X		X	X	X	X	X	X	X	X
PROMs		X			X		X	X	X	X	X

For further detail of the participation flow see trial schema (Figure 1).

Adverse Reaction (ARs), Serious Adverse Reactions (SARs), Related Unexpected Serious Adverse Events (RUSAEs) and Serious Adverse Events of Interest (SAEoI) will be collected from randomisation until the 24-month follow-up point. SAEoI are events identified by the research team that need additional monitoring; including angina/myocardial infarction, pulmonary embolism, serious skin reactions.

For peer review only

Data will be collected from all randomised participants, irrespective of treatment compliance. Where possible, endpoint and PROMs data will be collected from withdrawn participants who do not withdraw consent from further data collection.

Treatment and response assessment quality assurance

The trial will implement robust quality assurance of radiotherapy treatment and the primary endpoint assessments. The radiotherapy quality assurance (QA) programme will be implemented by the National Radiotherapy Trials Quality Assurance Group to ensure treatment is planned and delivered according to the trial protocol. All radiotherapy centres will complete pre-treatment benchmark exercises. Anonymised pre-treatment, 3-month and 6-month endoscopic photographs and MRI scans will be collected for all patients, with the first 2 patients from each site reviewed centrally. Trial Management Group (TMG) specialist members will ensure the inclusion/exclusion criteria have been met (baseline scan) and response has been correctly reported (3 and 6 months), providing feedback where necessary. Further review may be performed in batches.

Statistical analysis

A full statistical analysis plan will be written before any analyses are undertaken. No formal interim analyses are planned within the trial.

The primary endpoint will be assessed on an Modified *Intention to treat (MITT) population*; all randomised participants that have had at least one dose of trial treatment, grouped by the treatment arm to which they were randomised (regardless of ineligibility, non-compliance or withdrawal) [39].

A multivariable logistic regression model, adjusted for the minimisation factors, will be used to compare the proportion of participants achieving a cCR at 6-months. Significance testing will be 1-sided at the 20% level. The treatment effect will be presented as an odds ratio with a two-sided

For peer review only

60% confidence interval (equivalent to a 1-sided 80% lower confidence limit). Patterns of missing data and imputation methods (including multiple imputation) will be explored, if appropriate. A sensitivity analysis will be performed using the alternative definition of cCR.

All secondary analyses will use a 5% significance level and a two-sided confidence interval, where appropriate. Safety data will be presented on the *safety population*; participants are summarised on the treatment received regardless of randomisation. All other data will be summarised on an MITT population.

Summary statistics will be presented for toxicities on the number and proportion of participants experiencing ARs, SARs, RUSAEs, and SAEoI. Treatment compliance to the allocated radiotherapy (+/- chemotherapy) will be monitored and presented, including summaries for delays, dose modifications and discontinuation. PROMs will be summarised and reported using standard guidelines [32, 40, 41]. Mixed effects models will be used to assess differences between treatment arms. Cumulative incidence will be used to compare the time to stoma formation between treatment arms. Overall survival will be compared using the Cox Proportional Hazards model. Kaplan-Meier curves and survival estimates with corresponding confidence intervals will also be presented.

Trial organisation

Trial coordination, data management and statistical analysis will be directed and conducted by the trial specific project team at the University of Leeds CTRU. Trial supervision will be established according to the principles of Good Clinical Practice and in line with the relevant Research Governance Framework within the UK and CTRU standard operating procedures.

For peer review only

351 **Data collection and management**

352 Participating sites will record participant data on trial-specific case report forms and submit them to the CTRU. Participants will complete PROMs on the relevant paper questionnaire forms at the time of their clinic appointment. All trial data will be entered, validated and stored securely by CTRU. Missing and discrepant data will be flagged and additional data validations raised, as appropriate, from the CTRU data management team. Missing data (except PROMs) will be followed-up until received, or confirmed as not available. Data items regarding consent, patient safety and the primary endpoint will be subject to manual priority checking.

360 Data will be stored securely at Leeds CTRU. Only CTRU will have access to the data, prior to analysis and release of trial results. Upon completion of the trial, data will be stored in the sponsor archiving facility for a minimum of 15 years. After the final trial results have been published, interested researchers may contact the TMG and CTRU to request access to relevant data. Any requests will be reviewed by the TMG.

366 **Trial monitoring**

367 The TMG will provide ongoing clinical, practical and statistical advice on trial-related matters. The trial will be overseen by an independent Trial Steering Committee (TSC) and Data Monitoring and Ethics Committee (DMEC). The DMEC comprises two clinical oncologists and one statistician. They will review and monitor accumulating interim safety data and unblinded reports, at least annually. Their role is to protect the safety of the participants and maintain the research integrity of the study, advising the TSC on trial developments, including advice on trial continuation. A trial-specific DMEC charter has been developed in line with DAMOCLES [42, 43]. The sponsor and TSC have ultimate oversight over the conduct and continuation of the trial.

376 **Patient and Public Involvement**

For peer review only

APHRODITE has been developed with patient and public involvement (PPI) representatives from the earliest stages. Multiple aspects of both trial design and delivery have been shaped in collaboration with our PPI representatives, including the acceptability of the intervention, randomisation and the allocation ratio (2:1). One PPI representative was a co-applicant on the grant application (MJ). Two PPI representatives are members of the TMG (MJ, AB), and have provided patient perspective throughout set-up and recruitment. Upon their suggestion, APHRODITE has a patient-facing trial website, and a patient newsletter will be issued at regular intervals, with updates of trial progress and results when available. Trial feedback and communication with participants has been highlighted as a key importance by our PPI representatives.

Patient preference Sub-study

Dose escalation is associated with a greater probability of tumour control at the cost of greater risk of toxicity, yet little information exists as to what risks patients are willing to accept for a better chance of tumour control. The APHRODITE patient preference study (Chief Investigator, Dr Edward Webb), to be conducted alongside the main trial, aims to address this knowledge gap.

The patient preference sub-study uses a well-established survey technique called a discrete choice experiment (DCE) [44]. DCEs have a simple response format in which patients make a series of choices between hypothetical treatments characterised by attributes, such as probability of tumour control after two years and the risk of side effects [45]. The attribute levels change in each question, allowing the trade-offs participants make to be analysed [46]. The survey has been developed using qualitative methods in line with good practice recommendations [47]. Analysis of responses will quantify maximum acceptable risk of toxicity for a given probability of tumour control, as well as their preferences for process attributes, such as the number of treatment sessions and after-treatment support. Participants will complete the survey before treatment and six months after treatment. This will allow us to assess whether preferences are systematically influenced by treatment experience.

For peer review only

The sub-study plans to recruit 100-200 participants from sites participating in the APHRODITE trial, including APHRODITE participants, patients who decline APHRODITE participation, in addition to patients who are being managed non-surgically but do not meet the trial inclusion/exclusion criteria. Separate ethical approval has been obtained for the patient preference study from an NHS research ethics committee (reference 19/NE/0249).

Sample collection

Pre-treatment routine diagnostic formalin fixed paraffin embedded tumour biopsy tissue samples will be collected on all patients and sent to the University of Leeds, for storage. Consent will be taken for their use in future translational research. The routine glass Haematoxylin & Eosin stained slides used for diagnosis will be scanned to create a digital pathology resource for QA of the trial and support translational research, including research examining biomarkers of radiosensitivity and radioresistance.

ETHICS AND DISSEMINATION

The trial obtained ethical approval from North West Greater Manchester East research ethic committee (reference no. 19/NW/0565) and is registered in the ISRCTN registry (registration number 16158514). Confirmed by the UK Medicines and Healthcare products Regulation Agency, APHRODITE is not a trial of an investigational medicinal product (non-CTIMP). The trial is currently adhering to protocol version 2.0 (approved 31/03/2020).

The primary endpoint and early toxicity data will be analysed and reported when the last participant reaches 6-months post start of treatment. Further analyses will be conducted when all patients have reached 24-months follow-up. The final trial publication will be written by the TMG, and will adhere to ICMJE guidelines.

For peer review only

DISCUSSION

There is currently a lack of prospective trials of dedicated non-surgical management strategies in rectal cancer. It is therefore unclear whether increased radiotherapy dose may contribute to short-term tumour response, long-term local control and organ preservation. In particular, the lack of randomised data from the early phase setting has made it difficult to elucidate whether the high response rates observed in phase II trials are due to patient selection or treatment. The APHRODITE trial will provide high-level evidence whether dose escalation increases the cCR rate when directly compared to standard dose. Further studies will be required to provide evidence for whether a higher radiotherapy dose can provide improved long-term local control without surgery.

Other ongoing studies are examining dedicated organ preservation strategies for early rectal cancer. The STAR-TReC phase III trial (ISRCTN14240288) randomises patients with early (T1-3bN0) cancer with a preference for organ preservation between short- and long-course standard (chemo-)radiotherapy, with the aim of non-surgical management or local excision only. Both trials remain complimentary: STAR TREC uses standard radiotherapy in a patient population suitable for surgery, while APHRODITE will assess intensified treatment in an alternative patient group. Three other studies of radiotherapy dose escalation strategies for organ preservation are ongoing: The international OPERA phase III trial (NCT02505750) randomises patients between moderately dose escalated CRT (54Gy) and standard dose CRT (45Gy) followed by Papillon contact X-ray therapy (90Gy in 3 fractions); the single-centre randomised phase II MORPHEUS trial (NCT03051464) randomises patients between moderately dose escalated CRT (54Gy) and CRT (45Gy) followed by brachytherapy (30Gy in 3 fractions); and the Danish multi-centre WW3 phase II trial (NCT04095299) randomises patients between dose-escalated CRT (62Gy) and standard dose CRT (50.4Gy). All these studies require suitability for surgery, and the first two depend on

access to specialised treatment equipment. The APHRODITE study thus fills a clear research gap in the current international trial portfolio.

Studies of non-surgical management and organ preservation for rectal cancer have focused on clinical endpoints, e.g. cCR rate, stoma rate, and overall survival, as well as the clinicians' interpretation of patient outcome. There is a paucity of toxicity data including PROMs following nonsurgical management, particularly in a patient population usually excluded from clinical trials. The APHRODITE study will prospectively measure toxicity and PROMs, providing a valuable and unique dataset.

At present, there is a lack of understanding of how patients weigh and prioritise different potential outcomes, including the balance between efficacy and toxicity, in the setting of non-surgical rectal cancer treatment. The decision regarding which severity of treatment-related toxicities is acceptable for a given organ preservation strategy has previously been evaluated purely by clinicians. Two small studies examined patient preferences for organ-preserving and surgical treatment approaches [48, 49]. However, no studies have focused specifically on patient attitudes to treatment characteristics and outcomes following non-surgical management. The APHRODITE patient preference sub-study will help inform future dose escalation studies and help non-surgical management of rectal cancer to better reflect patient perspectives.

The APHRODITE trial will provide valuable, high quality, patient-centred evidence for escalated dose radiotherapy in rectal cancer patients not suitable for radical surgery, addressing an important research gap.

For peer review only

AUTHOR CONTRIBUTIONS

Contributors to the conception and design: AA, DSM, SB, SG.

Development of the protocol, trial documents and patient information sheet: AA, AB, AG, AS, AW, CB, DSM, DH, EH, JS, MJ, MS, NW, RA, RK, SB, SG, SN.

Writing manuscript: EH, AA.

Reviewed manuscript: AB, AG, AS, AW, CB, DSM, EW, MH, MS, MT, NW, SB, SPB, SG.

All authors read and approved the final manuscript.

FUNDING, SPONSOR AND COORDINATION

APHRODITE is supported by Yorkshire Cancer Research (award number L411). The study is coordinated by Leeds CTRU and sponsored by University of Leeds (GA18/118305). For enquires trial contact *email: APHRODITE@leeds.ac.uk*. Trial website: https://ctru.leeds.ac.uk/aphrodite/.

Both the sponsor and funder have no contribution to the study design and concept; collection, management, analysis, and interpretation of data; the writing of and decision to submit reports for publication. The sponsor and funder will review all manuscripts prior to publication.

COMPETING INTERESTS

EW reports grants from Yorkshire Cancer Research, during the conduct of the study.

MS reports personal fees from Servier, personal fees from Amgen, personal fees from Marck, outside the submitted work.

NW reports grants from Yorkshire Cancer Research, during the conduct of the study; grants from Cancer Research UK, outside the submitted work.

For peer review only

EXCLUSIVE LICENCE

I, the Submitting Author has the right to grant and does grant on behalf of all authors of the Work (as defined in the below author licence), an exclusive licence and/or a non-exclusive licence for contributions from authors who are: i) UK Crown employees; ii) where BMJ has agreed a CC-BY licence shall apply, and/or iii) in accordance with the terms applicable for US Federal Government officers or employees acting as part of their official duties; on a worldwide, perpetual, irrevocable, royalty-free basis to BMJ Publishing Group Ltd ("BMJ") its licensees and where the relevant Journal is co-owned by BMJ to the co-owners of the Journal, to publish the Work in BMJ Open and any other BMJ products and to exploit all rights, as set out in our licence.

The Submitting Author accepts and understands that any supply made under these terms is made by BMJ to the Submitting Author unless you are acting as an employee on behalf of your employer or a postgraduate student of an affiliated institution which is paying any applicable article publishing charge ("APC") for Open Access articles. Where the Submitting Author wishes to make the Work available on an Open Access basis (and intends to pay the relevant APC), the terms of reuse of such Open Access shall be governed by a Creative Commons licence – details of these licences and which Creative Commons licence will apply to this Work are set out in our licence referred to above.

FIGURE LEGEND: Figure 1 - *Trial Schema*

FIGURE LEGEND: Figure 2 - *Example treatment plans for the control arm (top panels, uniform 50.4 Gy in 28 fractions to the primary tumour and elective mesorectal volume); and intervention arm (lower panels, 62 Gy in 28 fractions to the primary tumour, 50.4Gy to the elective mesorectal volume), for a single patient.*

For peer review only

REFERENCES

1. CRUK. *Bowel cancer statistics*. 2017 [cited 2020; Available from: https://www.cancerresearchuk.org/health-professional/cancer-statistics/incidence.
2. Bentrem, D.J., et al., *T1 adenocarcinoma of the rectum: transanal excision or radical surgery?* Annals of surgery, 2005. **242**(4): p. 472.
3. Endreseth, B.H., et al., *Transanal excision vs. major surgery for T1 rectal cancer.* Diseases of the colon & rectum, 2005. **48**(7): p. 1380-1388.
4. Peeters, K.C., et al., *The TME trial after a median follow-up of 6 years: increased local control but no survival benefit in irradiated patients with resectable rectal carcinoma.* Annals of surgery, 2007. **246**(5): p. 693-701.
5. Sebag-Montefiore, D., et al., *Preoperative radiotherapy versus selective postoperative chemoradiotherapy in patients with rectal cancer (MRC CR07 and NCIC-CTG C016): a multicentre, randomised trial.* The Lancet, 2009. **373**(9666): p. 811-820.
6. *National Bowel Cancer Audit Annual Report 2020*. 2020.
7. Kapiteijn, E., et al., *Preoperative Radiotherapy Combined with Total Mesorectal Excision for Resectable Rectal Cancer.* New England Journal of Medicine, 2001. **345**(9): p. 638-646.
8. Tekkis, P.P., et al., *Operative mortality in colorectal cancer: prospective national study.* Bmj, 2003. **327**(7425): p. 1196-1201.
9. Rutten, H.J., et al., *Controversies of total mesorectal excision for rectal cancer in elderly patients.* The lancet oncology, 2008. **9**(5): p. 494-501.
10. Bakx, R., et al., *Morbidity of temporary loop ileostomies.* Digestive surgery, 2004. **21**(4): p. 277-281.
11. Habr-Gama, A., et al., *Complete clinical response after neoadjuvant chemoradiation therapy for distal rectal cancer: characterization of clinical and endoscopic findings for standardization.* Diseases of the Colon & Rectum, 2010. **53**(12): p. 1692-1698.

For peer review only

12. Van Der Valk, M.J., et al., *Long-term outcomes of clinical complete responders after neoadjuvant treatment for rectal cancer in the International Watch & Wait Database (IWWD): an international multicentre registry study.* The Lancet, 2018. **391**(10139): p. 2537-2545.

13. Renehan, A.G., et al., *Watch-and-wait approach versus surgical resection after chemoradiotherapy for patients with rectal cancer (the OnCoRe project): a propensity-score matched cohort analysis.* The Lancet Oncology, 2016. **17**(2): p. 174-183.

14. Sammour, T., et al., *Nonoperative management or 'watch and wait'for rectal cancer with complete clinical response after neoadjuvant chemoradiotherapy: a critical appraisal.* Annals of surgical oncology, 2017. **24**(7): p. 1904-1915.

15. Appelt, A.L., et al., *Radiation dose-response model for locally advanced rectal cancer after preoperative chemoradiation therapy.* International Journal of Radiation Oncology* Biology* Physics, 2013. **85**(1): p. 74-80.

16. Valentini, V., et al., *The INTERACT Trial: Long-term results of a randomised trial on preoperative capecitabine-based radiochemotherapy intensified by concomitant boost or oxaliplatin, for cT2 (distal)–cT3 rectal cancer.* Radiotherapy and Oncology, 2019. **134**: p. 110-118.

17. Appelt, A.L., et al., *High-dose chemoradiotherapy and watchful waiting for distal rectal cancer: a prospective observational study.* The Lancet oncology, 2015. **16**(8): p. 919-927.

18. Dizdarevic, E., et al., *Long-term patient-reported outcomes after high-dose chemoradiation therapy for nonsurgical management of distal rectal cancer.* International Journal of Radiation Oncology* Biology* Physics, 2020. **106**(3): p. 556-563.

19. Jensen, L.H., et al., *400MO Curative chemoradiation for low rectal cancer: Early clinical outcomes from a multicentre phase II trial.* Annals of Oncology, 2020. **31**(Suppl. 4): p. S411.

For peer review only

20. Maas, M., et al., *Long-term outcome in patients with a pathological complete response after chemoradiation for rectal cancer: a pooled analysis of individual patient data.* The lancet oncology, 2010. **11**(9): p. 835-844.

21. Appelt, A.L. and D. Sebag-Montefiore, *Technological advances in radiotherapy of rectal cancer: opportunities and challenges.* Current opinion in oncology, 2016. **28**(4): p. 353-358.

22. Roels, S., et al., *Definition and delineation of the clinical target volume for rectal cancer.* International Journal of Radiation Oncology* Biology* Physics, 2006. **65**(4): p. 1129-1142.

23. Leibold, T., et al., *Prognostic implications of the distribution of lymph node metastases in rectal cancer after neoadjuvant chemoradiotherapy.* Journal of clinical oncology, 2008. **26**(13): p. 2106-2111.

24. Nijkamp, J., et al., *Three-dimensional analysis of recurrence patterns in rectal cancer: the cranial border in hypofractionated preoperative radiotherapy can be lowered.* International Journal of Radiation Oncology* Biology* Physics, 2011. **80**(1): p. 103-110.

25. Appelt, A.L., et al., *Robust dose planning objectives for mesorectal radiotherapy of early stage rectal cancer–A multicentre dose planning study.* Technical Innovations & Patient Support in Radiation Oncology, 2019. **11**: p. 14-21.

26. Peters, F.P., et al., *Mesorectal radiotherapy for early stage rectal cancer: A novel target volume.* Clinical and Translational Radiation Oncology, 2020. **21**: p. 104-111.

27. Patel, U.B., et al., *Magnetic resonance imaging–detected tumor response for locally advanced rectal cancer predicts survival outcomes: MERCURY experience.* Journal of Clinical Oncology, 2011. **29**(28): p. 3753-3760.

28. Patel, U.B., et al., *MRI after treatment of locally advanced rectal cancer: how to report tumor response—the MERCURY experience.* American Journal of Roentgenology, 2012. **199**(4): p. W486-W495.

For peer review only

29. Fokas, E., et al., *International consensus recommendations on key outcome measures for organ preservation after (chemo) radiotherapy in patients with rectal cancer.* Nature Reviews Clinical Oncology, 2021: p. 1-12.

30. Aaronson, N.K., et al., *The European Organization for Research and Treatment of Cancer QLQ-C30: a quality-of-life instrument for use in international clinical trials in oncology.* J Natl Cancer Inst, 1993. **85**(5): p. 365-76.

31. Gujral, S., et al., *Assessing quality of life in patients with colorectal cancer: an update of the EORTC quality of life questionnaire.* European journal of cancer, 2007. **43**(10): p. 1564-1573.

32. Emmertsen, K.J. and S. Laurberg, *Low anterior resection syndrome score: development and validation of a symptom-based scoring system for bowel dysfunction after low anterior resection for rectal cancer.* Annals of surgery, 2012. **255**(5): p. 922-928.

33. Dossa, F., et al., *A watch-and-wait approach for locally advanced rectal cancer after a clinical complete response following neoadjuvant chemoradiation: a systematic review and meta-analysis.* The Lancet Gastroenterology hepatology, 2017. **2**(7): p. 501-513.

34. Appelt, A.L., et al., *Radiation dose-response model for locally advanced rectal cancer after preoperative chemoradiation therapy.* International Journal of Radiation Oncology Biology Physics, 2013. **85**(1): p. 74-80.

35. Appelt, A.L. and A. Jakobsen, *Radiation techniques for increasing local control in the non-surgical management of rectal cancer.* Current Colorectal Cancer Reports, 2015. **11**(5): p. 267-274.

36. Rubinstein, L.V., et al., *Design issues of randomized phase II trials and a proposal for phase II screening trials.* Journal of Clinical Oncology, 2005. **23**(28): p. 7199-7206.

37. Korn, E.L., et al., *Design issues in randomized phase II/III trials.* Journal of Clinical Oncology, 2012. **30**(6): p. 667.

For peer review only

38. Ratain, M.J. and D. Sargent, *Optimising the design of phase II oncology trials: the importance of randomisation.* European journal of cancer, 2009. **45**(2): p. 275-280.

39. Abraha, I. and A. Montedori, *Modified intention to treat reporting in randomised controlled trials: systematic review.* Bmj, 2010. **340**.

40. Calvert, M., et al., *Reporting of patient-reported outcomes in randomized trials: the CONSORT PRO extension.* Jama, 2013. **309**(8): p. 814-822.

41. Fayers, P., et al., *EORTC QLQ–C30 scoring manual.* 1995: European Organisation for Research and Treatment of Cancer.

42. DAMOCLES Study Group, *A proposed charter for clinical trial data monitoring committees: helping them to do their job well.* The Lancet, 2005. **365**(9460): p. 711-722.

43. MRC Clinical Trials Unit, *Template IDMC Charter version 2.01.* 2006.

44. Soekhai, V., et al., *Discrete choice experiments in health economics: past, present and future.* Pharmacoeconomics, 2019. **37**(2): p. 201-226.

45. Ryan, M., *Discrete choice experiments in health care*. 2004, British Medical Journal Publishing Group.

46. Lancsar, E., D.G. Fiebig, and A.R. Hole, *Discrete choice experiments: a guide to model specification, estimation and software.* Pharmacoeconomics, 2017. **35**(7): p. 697-716.

47. Coast, J., et al., *Using qualitative methods for attribute development for discrete choice experiments: issues and recommendations.* Health economics, 2012. **21**(6): p. 730-741.

48. Couwenberg, A.M., et al., *Utility scores and preferences for surgical and organ-sparing approaches for treatment of intermediate and high-risk rectal cancer.* Diseases of the Colon & Rectum, 2018. **61**(8): p. 911-919.

49. Gani, C., et al., *Organ preservation in rectal cancer: The patients' perspective.* Frontiers in oncology, 2019. **9**: p. 318.

For peer review only

Multi-disciplinary team deems patient unsuitable for radical TME resection of their tumour either because they are considered to be at increased surgical risk or they would find it difficult to manage a stoma.

Eligibility criteria

Biopsy proven non-metastatic rectal adenocarcinoma; ECOG PS 0-2
Primary tumour T1-T3b (T3b: 1-5mm spread beyond muscularis propria)
N0 or Nx on pelvic MRI; Maximum tumour diameter 4cm
Maximum height of superior aspect of tumour is the S2-3 junction

Randomise 1:2 (104 patients total)

Standard dose CRT 50.4 Gy in 28 fractions to mesorectal volume treating once daily Mon-Fri over 5.5 weeks using IMRT, with concurrent fluoropyrimidine chemotherapy*

Escalated dose CRT 50.4 Gy to mesorectal volume, with boost to total 62 Gy to tumour, in 28 fractions treating once daily Mon-Fri over 5.5 weeks using synchronous integrated boost IMRT, with concurrent fluoropyriidine chemotherapy*

Follow-up Schedule (post-start of CRT)

7.5 weeks (2 weeks post CRT completion): Phone follow-up for toxicity (CTCAE v5.0) and Performance Status (PS)
3m: Clinical exam, PS and digital rectal exam (DRE), flexible sigmoidoscopy, MRI pelvis, toxicity, PROM (EORTC C30, EORTC CR29, LARS score)
6m: Clinical exam, PS, DRE, flexible sigmoidoscopy, MRI pelvis, toxicity, PROM
9m: Clinical exam, PS, DRE, toxicity, PROM
12m: Clinical exam, PS, DRE, flexible sigmoidoscopy, toxicity, PROM
24m: Clinical exam, PS, DRE toxicity, PROM

Primary end point: Clinical complete response at 6 months

Secondary end points: Acute toxicity, treatment compliance, late toxicity, patient reported outcome measures (PROMs) including health-related quality of life (HRQoL), colostomy rate, overall survival

*At treating team discretion, concurrent chemotherapy can be used either at 75% dose or omitted completely. This choice must be declared prior to randomisation

For peer review only - http://bmjopen.bmj.com/site/about/guidelines.xhtml

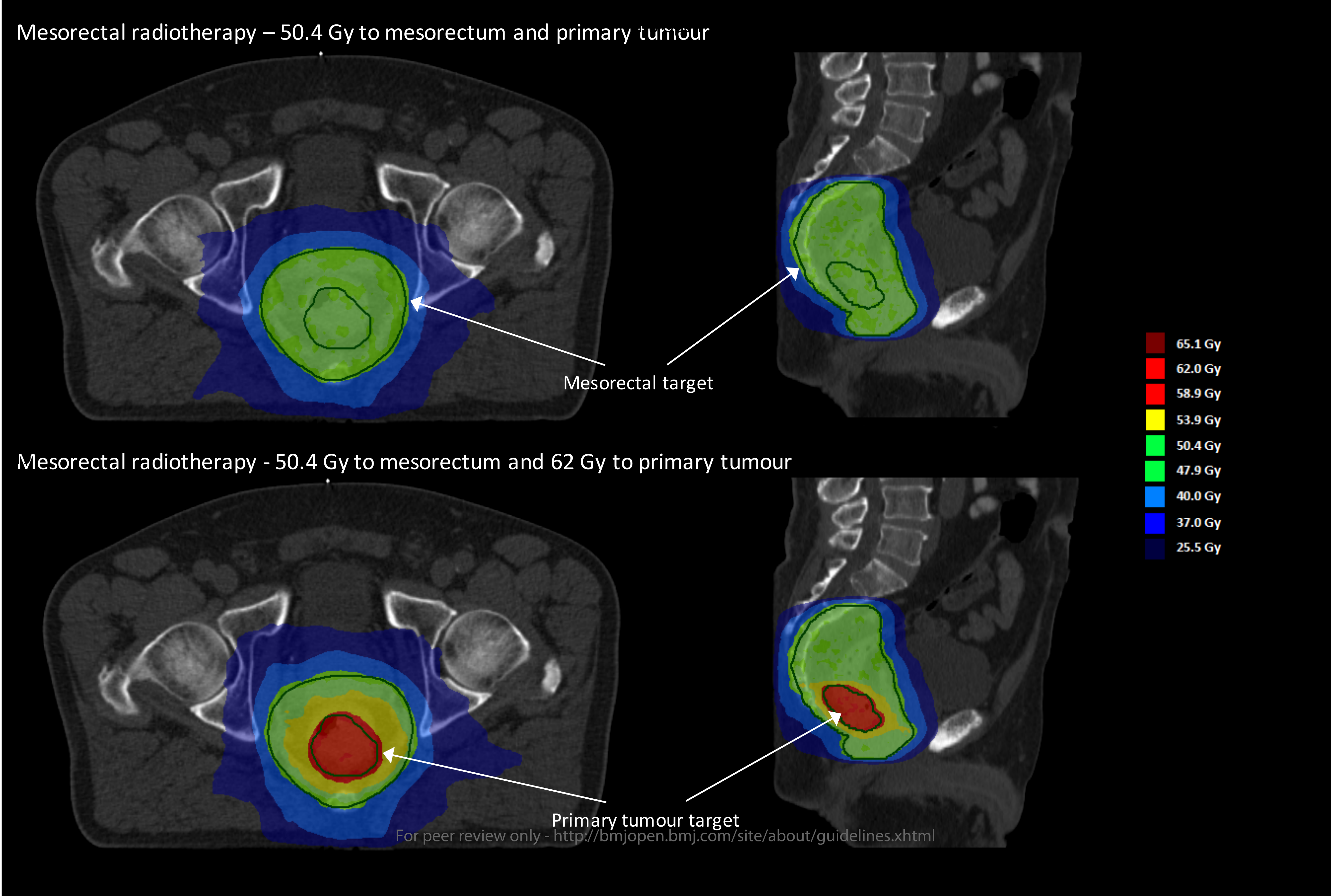

For peer review only - http://bmjopen.bmj.com/site/about/guidelines.xhtml

Delete this line, then print on Trust/Hospital headed paper

Participant ID:	Initials:
Date of Birth:	NHS/Hospital Number:
ISRCTN: 161585514	Principal Investigator:

APHRODITE

SAMPLE

For peer review only

PARTICIPANT CONSENT FORM

Please initial each box

1. I confirm that I have read and understand the information sheet for the above study and have had the opportunity to ask questions.
2. I understand that my participation in this study is voluntary and that I am free to withdraw at any time without my medical care or legal rights being affected. I understand that even if I withdraw from the above study, the data and samples collected from me will be used in analysing the results of the study and in some cases further information about any unwanted effects of my treatment may need to be collected by the study team.
3. I understand that my healthcare records may be looked at by authorised individuals from the study team, regulatory bodies or Sponsor in order to check that the study is being carried out correctly.
4. I agree to a copy of this Consent Form being sent to the Clinical Trials Research Unit (CTRU).
5. I will allow any information or results arising from this study to be used for healthcare and/or further medical research upon the understanding that my identity will remain anonymous wherever possible.
6. I will allow any information or results arising from this study to be shared anonymously with researchers at the University of Leeds conducting the study "How do patients make decisions about rectal cancer treatment?" I understand that my identity will remain anonymous.

7. I understand that if during this study my clinical care team determine that I have lost my ability to make my own decisions, no further study intervention will be given. I agree that the information collected up until this point will remain on file and will be included in the analysis.

8. I agree that my GP, or any other doctor treating me, will be notified of my participation in this study.

9. I agree to take part in the study.

The following points are OPTIONAL.

Please initial each box

	Yes	No
I give permission for surplus samples from my cancer biopsy that have been stored in the hospital pathology laboratory to be retrieved and used in the future for colorectal cancer research, including genetic research.		
I understand that my tissue sample is a 'gift' that may be used in future research that receives ethical approval. I understand that my sample and data collected from it may be shared on a collaborative basis with researchers in the UK and potentially, centres abroad, including outside the European Economic Area.		
I give permission that my endoscopy photos, radiotherapy data, CT and MRI scans may be used in future research. I understand that my radiotherapy data scans may be shared on a collaborative basis with researchers in the UK and potentially, centres, abroad, including outside the European Economic Area.		

SAMPLE

For peer review only

Patient:

Signature…………………………………………………………………………

Name(block capitals)……….………………………………………….……………………

Date…………………………………………….…………………………………

Investigator:

I have explained the study to the above named patient and he/she has indicated his/her willingness to participate.

Signature………………………………………..…………………………………

Name(block capitals)………………….……………………………………….…………………

Date……………………………………………………………………………………

(If used) Witness:

Signature………………………………………..…………………………………

Name(block capitals)…………………………………………….……………………….….

Date…………………………………………….…………………………………

(1 copy for patient; 1 for the CTRU; 1 held in patient notes, original stored in Investigator Site File)

SAMPLE

For peer review only

STANDARD PROTOCOL ITEMS: RECOMMENDATIONS FOR INTERVENTIONAL TRIALS

SPIRIT 2013 Checklist: Recommended items to address in a clinical trial protocol and related documents*

Section/item	Item No	Description	Addressed on page number
**Administrative information**			
Title	1	Descriptive title identifying the study design, population, interventions, and, if applicable, trial acronym	1 ___________
Trial registration	2a	Trial identifier and registry name. If not yet registered, name of intended registry	4, 19__________
	2b	All items from the World Health Organization Trial Registration Data Set	Yes __________
Protocol version	3	Date and version identifier	20 ___________
Funding	4	Sources and types of financial, material, and other support	23 ___________
Roles and responsibilities	5a	Names, affiliations, and roles of protocol contributors	1, 23 _________
	5b	Name and contact information for the trial sponsor	23 ___________
	5c	Role of study sponsor and funders, if any, in study design; collection, management, analysis, and interpretation of data; writing of the report; and the decision to submit the report for publication, including whether they will have ultimate authority over any of these activities	23 ___________
	5d	Composition, roles, and responsibilities of the coordinating centre, steering committee, endpoint adjudication committee, data management team, and other individuals or groups overseeing the trial, if applicable (see Item 21a for data monitoring committee)	17, 18 ________

For peer review only

**Introduction**			
Background and rationale	6a	Description of research question and justification for undertaking the trial, including summary of relevant studies (published and unpublished) examining benefits and harms for each intervention	6,7,8_________
	6b	Explanation for choice of comparators	14__________
Objectives	7	Specific objectives or hypotheses	9,10 ________
Trial design	8	Description of trial design including type of trial (e.g., parallel group, crossover, factorial, single group), allocation ratio, and framework (e.g., superiority, equivalence, non-inferiority, exploratory)	8,9 _________
**Methods: Participants, interventions, and outcomes**			
Study setting	9	Description of study settings (e.g., community clinic, academic hospital) and list of countries where data will be collected. Reference to where list of study sites can be obtained	8,9_______
Eligibility criteria	10	Inclusion and exclusion criteria for participants. If applicable, eligibility criteria for study centres and individuals who will perform the interventions (e.g., surgeons, psychotherapists)	12, Table 1 __
Interventions	11a	Interventions for each group with sufficient detail to allow replication, including how and when they will be administered	14_______
	11b	Criteria for discontinuing or modifying allocated interventions for a given trial participant (e.g., drug dose change in response to harms, participant request, or improving/worsening disease)	________
	11c	Strategies to improve adherence to intervention protocols, and any procedures for monitoring adherence (e.g., drug tablet return, laboratory tests)	18 _______
	11d	Relevant concomitant care and interventions that are permitted or prohibited during the trial	12 _______
Outcomes	12	Primary, secondary, and other outcomes, including the specific measurement variable (e.g., systolic blood pressure), analysis metric (e.g., change from baseline, final value, time to event), method of aggregation (e.g., median, proportion), and time point for each outcome. Explanation of the clinical relevance of chosen efficacy and harm outcomes is strongly recommended	9, 10 ____
Participant timeline	13	Time schedule of enrolment, interventions (including any run-ins and washouts), assessments, and visits for participants. A schematic diagram is highly recommended (see Figure)	Table 2, Figure 1

For peer review only - http://bmjopen.bmj.com/site/about/guidelines.xhtml

Sample size	14	Estimated number of participants needed to achieve study objectives and how it was determined, including clinical and statistical assumptions supporting any sample size calculations	13, 14_______
Recruitment	15	Strategies for achieving adequate participant enrolment to reach target sample size	13 ______

Methods: Assignment of interventions (for controlled trials)

Allocation:

Sequence generation	16a	Method of generating the allocation sequence (e.g., computer-generated random numbers), and list of any factors for stratification. To reduce predictability of a random sequence, details of any planned restriction (eg, blocking) should be provided in a separate document that is unavailable to those who enrol participants or assign interventions	13 __________
Allocation concealment mechanism	16b	Mechanism of implementing the allocation sequence (e.g., central telephone; sequentially numbered, opaque, sealed envelopes), describing any steps to conceal the sequence until interventions are assigned	13 ___________
Implementation	16c	Who will generate the allocation sequence, who will enrol participants, and who will assign participants to interventions	13 _________
Blinding (masking)	17a	Who will be blinded after assignment to interventions (e.g., trial participants, care providers, outcome assessors, data analysts), and how	N/A _________
	17b	If blinded, circumstances under which unblinding is permissible, and procedure for revealing a participant's allocated intervention during the trial	N/A__________

Methods: Data collection, management, and analysis

Data collection methods	18a	Plans for assessment and collection of outcome, baseline, and other trial data, including any related processes to promote data quality (e.g., duplicate measurements, training of assessors) and a description of study instruments (e.g., questionnaires, laboratory tests) along with their reliability and validity, if known. Reference to where data collection forms can be found, if not in the protocol	15, (Figure 1), (Table 2)
	18b	Plans to promote participant retention and complete follow-up, including list of any outcome data to be collected for participants who discontinue or deviate from intervention protocols	16, 18________

Data management	19	Plans for data entry, coding, security, and storage, including any related processes to promote data quality (e.g., double data entry; range checks for data values). Reference to where details of data management procedures can be found, if not in the protocol	18 _________
Statistical methods	20a	Statistical methods for analysing primary and secondary outcomes. Reference to where other details of the statistical analysis plan can be found, if not in the protocol	16,17______
	20b	Methods for any additional analyses (e.g., subgroup and adjusted analyses)	16, 17_______
	20c	Definition of analysis population relating to protocol non-adherence (e.g., as randomised analysis), and any statistical methods to handle missing data (e.g., multiple imputation)	16, 17 ______
**Methods: Monitoring**			
Data monitoring	21a	Composition of data monitoring committee (DMC); summary of its role and reporting structure; statement of whether it is independent from the sponsor and competing interests; and reference to where further details about its charter can be found, if not in the protocol. Alternatively, an explanation of why a DMC is not needed	18_____
	21b	Description of any interim analyses and stopping guidelines, including who will have access to these interim results and make the final decision to terminate the trial	16 ________
Harms	22	Plans for collecting, assessing, reporting, and managing solicited and spontaneously reported adverse events and other unintended effects of trial interventions or trial conduct	15, 17 ____
Auditing	23	Frequency and procedures for auditing trial conduct, if any, and whether the process will be independent from investigators and the sponsor	_________
**Ethics and dissemination**			
Research ethics approval	24	Plans for seeking research ethics committee/institutional review board (REC/IRB) approval	20 __________
Protocol amendments	25	Plans for communicating important protocol modifications (e.g., changes to eligibility criteria, outcomes, analyses) to relevant parties (e.g., investigators, REC/IRBs, trial participants, trial registries, journals, regulators)	____________

Consent or assent	26a	Who will obtain informed consent or assent from potential trial participants or authorised surrogates, and how (see Item 32)	13 ____________
	26b	Additional consent provisions for collection and use of participant data and biological specimens in ancillary studies, if applicable	13, 20_________
Confidentiality	27	How personal information about potential and enrolled participants will be collected, shared, and maintained in order to protect confidentiality before, during, and after the trial	18 _________
Declaration of interests	28	Financial and other competing interests for principal investigators for the overall trial and each study site	23 ____________
Access to data	29	Statement of who will have access to the final trial dataset, and disclosure of contractual agreements that limit such access for investigators	18 _________
Ancillary and post-trial care	30	Provisions, if any, for ancillary and post-trial care, and for compensation to those who suffer harm from trial participation	N/A _________
Dissemination policy	31a	Plans for investigators and sponsor to communicate trial results to participants, healthcare professionals, the public, and other relevant groups (e.g., via publication, reporting in results databases, or other data sharing arrangements), including any publication restrictions	19 _____
	31b	Authorship eligibility guidelines and any intended use of professional writers	23 ___________
	31c	Plans, if any, for granting public access to the full protocol, participant-level dataset, and statistical code	18 __________
**Appendices**			
Informed consent materials	32	Model consent form and other related documentation given to participants and authorised surrogates	__________
Biological specimens	33	Plans for collection, laboratory evaluation, and storage of biological specimens for genetic or molecular analysis in the current trial and for future use in ancillary studies, if applicable	20___________

*It is strongly recommended that this checklist be read in conjunction with the SPIRIT 2013 Explanation & Elaboration for important clarification on the items. Amendments to the protocol should be tracked and dated. The SPIRIT checklist is copyrighted by the SPIRIT Group under the Creative Commons "Attribution-NonCommercial-NoDerivs 3.0 Unported" license.
